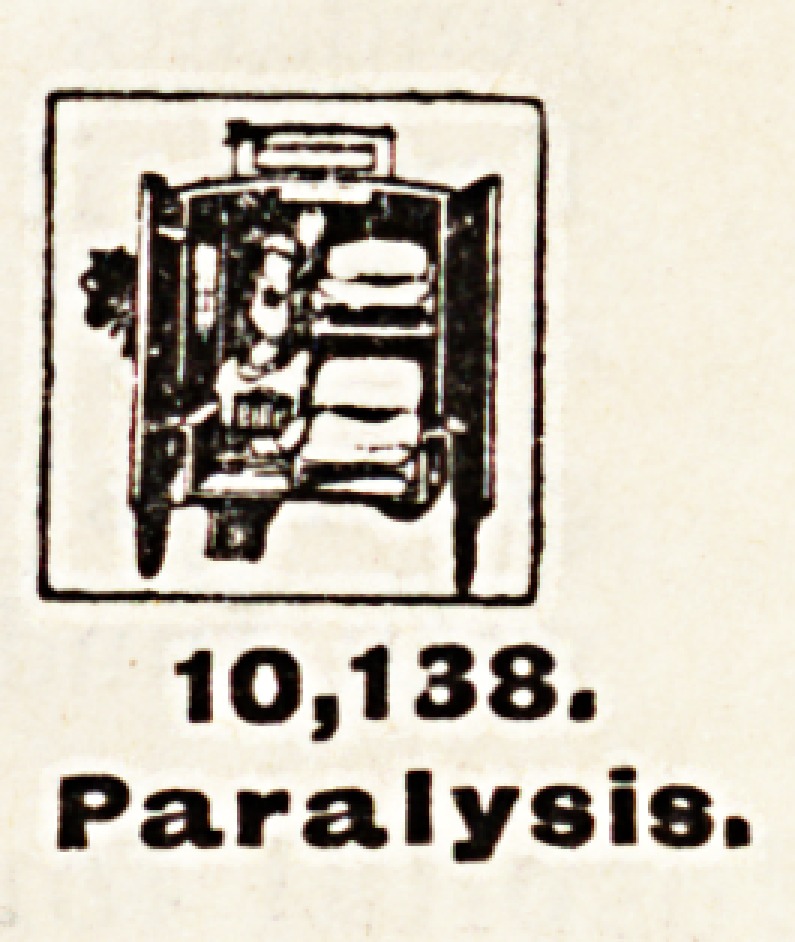# Hospital Sunday Special Number

**Published:** 1915-06-13

**Authors:** 


The Hospital.
Being The Hospital Sunday Special Number.

### WAR-TIME AT A VOLUNTARY HOSPITAL

### St. Thomas's Hospital as a Type.

The war has brought many changes in our lives, in our homes, and institutions, and nowhere has it been felt more than in the great voluntary hospitals of London. These have been affected in a multitude of ways. As might have been expected, the services of the distinguished members of the staff have been freely called upon for the benefit of the country. Nurses have been requisitioned for duty both in Navy and Army hospitals. Students have rushed off eagerly to serve their country, both qualified and unqualified men. Many of the lay staff just as enthusiastic have joined the combatant forces. With their reduced staffs all the hospitals have been called upon to undertake very heavy extra duty and to treat the wounded. It is invidious to single out any one hospital as doing more than others when all have done their best, but we may take St. Thomas's as a type and describe what has been done there in this grave time of emergency, and draw a very accurate conclusion as to what has been done, not only in the great hospitals in London, but in all similar institutions throughout the country.

Immediately on the outbreak of war the Governors wrote on August 5 and offered to place at least 200 beds for non-commissioned officers and men and thirty beds for officers at the service of either Navy or Army. The offer was gladly accepted, and five wards on the first floor were set apart for the purpose. A meeting of the staff was held, and they cordially endorsed the action taken by the Governors. They even went further, and it was suggested amongst them that at least twice as many beds might be offered for this noble purpose. The Governors, however, felt that it was their duty first and foremost not to reduce to a point of grave injustice the number of beds allotted to civilian cases. Examining the matter carefully, they came to the conclusion that they should take two men's surgical wards, one men's medical ward, one female surgical ward, and one female medical ward. To some extent this could be done owing to the large number of men who are being drafted into the Army and the slight reduction resulting therefrom of the number of men applying for admission and

*Underwood and Underwood*] British Soldiers in a Voluntary Hospital Ward. [*London*
(St. Thomas's Hospital.)

Underwood and Underwood] BRITISH WOUNDED AT ST. THOMAS'S HOSPITAL. [London.

### War-time at a Voluntary Hospital.

treatment in the hospital. Further, under the system of allowance there is no doubt whatever that both women and children are better off. Better feeding at home and better living have undoubtedly reduced somewhat the number of cases of women applying for treatment in the hospital. The wards of St. Thomas's are fine, large, airy wards, with abundant cubic space per bed and excellent ventilation. So far, therefore, as space was concerned there was no reason why the usual thirty beds should not be increased by at least one-third, and in times of heavy pressure and emergency the numbers of patients admitted have been increased 30 per cent. This could not be done without the nursing staff, under the lead of the able matron of St. Thomas's, being prepared to bear their extra burden. They have been assisted by voluntary nurses, some with experience, some anxious to learn all that was possible and to be of use to those who are fighting for them.

On September 3 the first batch of wounded arrived, 123 men and one officer. They were practically all men who had taken part in the great retreat from Mons, and had been drafted over as soon as possible, shipped from Havre to Southampton, and brought thence by train to Waterloo, where they were met by the well-organised ambulances, under the direction of Mr. Clinton Dent, of the City Red Cross Ambulance Association. Amongst these men, however, very few were wounded; mostly, they were suffering from the extraordinary hardships of the early days of the campaign. One man with a bullet through his foot was quite a notable character, and he had shot himself by accident. Nothing showed how keenly the country was stirred more than the great number of strangers who applied for permission to visit these soldiers. There was very great risk that visitors inexperienced in hospital work would be a source of considerable danger amongst these men. Discipline was only maintained with very considerable difficulty. All were heroes, and hero-worship without judgment may be a little dangerous to the heroes. Many visitors, however, came to see these men; their presence cheered them and served to convey a message to those still fighting at the Front of how deeply the best of our land were stirred and were heart and soul in sympathy with those risking life and limb in their country's cause.

Their Majesties the King and Queen paid a semi-private visit to the hospital on September 8. Strict orders were given that none but the actual officials of the hospital should meet their Majesties. They wanted only the Secretary and the Matron and the physicians and surgeons in charge of the various wards to tell them of the cases. Their wishes were naturally strictly carried out, and their Majesties spoke individually to practically every patient in the wards. In those days men came back battered and torn by the turmoil of the strife, scarcely any had badges. These had been secured as a rule by their sympathetic French admirers, and in reply to his Majesty's "Where is your badge?" it was invariably "Souvenir, Sir." Their Majesties' visit was followed by practically all the Royal Princesses, by the Secretary of State for War, and other distinguished persons.

The hospital has since carried on its work until, in the first nine months of the war, a total of 211 officers and 1,371 non-commissioned officers and men have been treated in the wards of St. Thomas's. In the early days the cases were, as a rule, comparatively slight in character. Army regulations, however, led to their being retained in the hospital long after civilian cases of a similar type would have been discharged, but at last came the time when our armies moving up from the Aisne, and starting from Hazebrouck, met the Germans on the Belgian frontier. Then much more severe cases began to arrive. Boulogne was used very frequently as the port of embarkation for the hospital ships. Still, some came to Southampton; many of those who went through the first great battle of Ypres were hurried across, almost straight from the battlefield, *via* Boulogne and Folkestone, to Charing Cross, and so on to the hospital. Many arrived within three days of being wounded, and the demands on the services of the staff were proportionately heavy. All such demands, however, were readily met.

Early in February the Government became fully alive to the fact that much increased accommodation for wounded was required in London. They beat about for means of housing the wounded and of staffing them. The new King George Hospital had been devised by the Red Cross Society and made widely known through the columns of the *Times*. Little, however, has been put before the public of what a deal of extra work our hospitals have willingly undertaken. In some instances the staffs have been invited to take over the medical and surgical care of patients established in infirmaries converted to military hospitals or to improvised military hospitals in national schools and other similar places. On application being made to St. Thomas's from the office of the D.D.M.S. of London a suggestion was put forward at once from the Governors that great economy might be secured with increased efficiency if extra accommodation could be found within the hospital walls, and it was suggested that huts should be built in each of the quadrangles between the blocks. The suggestion was warmly welcomed, and at last, after going through the usual routine, authority was received on March 31 for the Governors to proceed with the erection of these huts at the price submitted, and the War Office would bear the expense of erection. Now, at the end of May the building of these huts is completed, and progress has been made with their equipment. Already three huts are practically fit for use. There are six huts altogether for patients—two of sixty-six beds each, one of sixty beds, one of eighty beds, and two of thirty beds each. The two huts of thirty beds are what may be called single huts. The other four are divided by a spine 5 feet 6 inches high, with excellent cross ventilation, ample light, and are really charming wards. As may be imagined, there

### War-time at a Voluntary Hospital.

is a striking contrast between the hospital equipment and the War Office equipment, but the latter is at least serviceable, and we trust it will prove fully adequate to the end of the war. Each of these wards is provided with lavatories and bathrooms of the hospital type, each has its own little linen room, servery, and sister's small room. They are warmed by gas radiators, lighted by electricity, with abundant plugs for any hand-lights. In construction these wards, which are 104 feet long, 40 feet broad the double huts, 20 feet the single, 10 feet 6 inches high, are built of a wooden frame, outside asbestos slabs, inside fibrous plaster coloured with a pleasant light green. The window sashes open with a sort of fanlight, top and bottom. In addition, accommodation has been built for forty orderlies, for six sergeants, and a temporary extension of the kitchen has been made. The total cost is roughly £10,000.

When the Governors made their original offer they undertook to bear the full expense of the treatment of both officers and men, and this arrangement was maintained right through to March 1. The hospital resources were strained to almost breaking point, and when extra accommodation was asked for by the War Office they naturally looked for payment for maintenance. The agreement applies to all soldiers treated at the hospital, and the Governors will therefore receive payment at an agreed sum per diem from March 1. Though this amount is considerably less than the average cost of an ordinary hospital patient, it is felt that such help may be secured by the Governors in acknowledgment of the great work undertaken by them that the finances of the hospital may not be permanently impaired. Some donors have already recognised what is being done. Two American ladies in the early days of August expressed their anxiety to do everything in their power to help and to identify themselves with one hospital. Through one of the senior physicians of the hospital, Dr. Hector Mackenzie, these two ladies, Mrs. Cornelius Garrison and her sister, Miss Randell, sent a donation of £5,000 as a War Fund. That the men have appreciated their treatment is abundantly proved. Many friends of the hospital have generously sent supplies of cigarettes, tobacco, periodicals, flowers, and other gifts. The Colonial Governments have rendered very valuable aid by gifts of provisions, such as meat, fruit, flour, potatoes, etc.

One feature of the work must not be passed without mention. Quite a large number of young men who have applied for enlistment were found to be unsuitable unless they had an operation done for the cure of hernia, varicocele, and other small ailments. An average of something like forty per week have been taken in since the early days of the war. This could not have been done without generous aid from various convalescent homes and other institutions which have been specially prepared for the purpose. Lady Henry Somerset placed no fewer than fifty beds at Duxhurst at the disposal of the hospital. The Speaker gave timely aid at Wickham Market, and has taken a stream of patients—something like twenty—per week. Other homes have been opened by Mrs. Hamilton Grace, Mrs. Baxendale, and Mr. Lambert, and the great and generous work done deserves sincere thanks and appreciation from the nation.

A visit to the wards at St. Thomas's is convincing proof of the general happiness of the patients. The way in which the resident staff have risen to the occasion is worthy of all praise. They have generously undertaken an enormous amount of extra duty, and they have bravely stuck to their hospital work when their hearts led them to be away where they would love to be, with their colleagues abroad. They have still denied themselves and continued to do the routine hospital work, which cannot possibly be neglected.

### THE GOSPEL OF PERSONAL SERVICE.

Hospital Sunday is the classic example of an anniversary day specially set apart for the benefit of charity. Not only so, but in this year of war it is the outstanding example of an annual charitable function which there has been no suggestion to discard. The street collections in different localities, the charitable bazaars and garden-parties, the hospital festival dinner itself, have all been waived. The annual appeal to meet a deficiency, the special appeal to provide funds for some new extension of work, have mostly been scratched, like racing, as unsuited to the sentiment and stern necessity of the year. But Hospital Sunday is an exception. This one great day of appeal on behalf of the voluntary hospitals will be honoured for the fifty-sixth year in succession from every pulpit, even from those in which aspects of the war have threatened to oust the traditional messages of other festivals, Sundays, and red-letter days. Nor does this apparent exception to the fear that the claims of charity are not likely to be attended to in time of war require much explanation. Hospital Sunday is, in fact, the festival which symbolises the great idea which is beginning to electrify England—that idea which is the tradition of the voluntary hospitals, of personal service. The need for this has been stirring uneasily in the minds of Englishmen during the past nine months. The newspapers have fixed on an outward expression of it in the consideration which they have given to compulsory military service, and to the need which is also being urged for what is called the mobilisation of all our resources. This is the underlying reason why Hospital Sunday, alone among the festivals of charity, maintains its position this year when so many smaller examples have yielded to the circumstance of war. But, indeed, besides this, the ray of light and cheerfulness, the reconstructive activity which has been thrown into high relief amid all the ruin of destruction, has been the work which the voluntary hospitals have been doing for the wounded and the sick, the poisoned and disabled of the war. It is a great thought that the gospel of personal service which the voluntary system of hospital support has preached on Hospital Sunday through an almost undisturbed half-century of peace should have become a gospel for the veriest commercial newspaper reader in the present time of war. Small wonder, then, that the persistent, quiet voice of voluntary hospital teaching is become a clarion call, though, alas! it has required a European conflagration to illustrate the necessity for it.

Nowadays, then, the war rather than the voluntary hospital is compelling men's attention to the fact that personal service must be the mainspring of any man in every nation who values his life and its mission, as a high calling for which the existence of the individual citizen is only the period of office of a trustee. The gospel is on every man's lips; the work which the hospitals are now performing is before every man's eyes. The work which these institutions do, and the need for it, is more clearly realised by the general public than ever before. Its value is beyond dispute. On this occasion Hospital Sunday, therefore, has not so much to drive the lesson home as to remind the nation that an immense sum of money is necessary for the work of the voluntary hospitals to be carried on under the heavy imposition which is now being laid upon them. For once their example overshadows in the public eye the teaching which they exist so largely to inculcate; and so the public has merely to be reminded that the labourer is worthy of his hire. It would, indeed, be a strange and lamentable thing if this year's work, which has surpassed all previous efforts, should not result in a record collection on Hospital Sunday also. Imagine a vast public hall filled to overflowing with a crowd of people who have assembled to assist by founding a great fund, the cause of the widows and dependants of those fallen in the war, full of enthusiasm, cheering the speakers, applauding the resolutions—imagine this vast assembly so excited by good intentions as to break up having forgotten to call for any subscriptions or to pass round the hat. Such a situation is easily conceivable, and has probably happened before now, but the abysmal folly of the proceeding is exactly similar to what will happen this year if the total subscribed to the Hospital Sunday Fund does not beat all previous records. The public knows the facts about the hospitals, is full of enthusiasm on their behalf, is genuinely moved by the work which they are performing. But this knowledge, enthusiasm, and gratitude must be crystallised into a practical form, so that the good work may be maintained from day to day. Hospital Sunday exists so to crystallise it.

Our voluntary hospitals depend entirely on what has been, and still is, given voluntarily to them by way of donation, annual subscription, or legacy. It is true that the War Office is giving a sum round about 4s. a day to many, though not all, of the hospitals for every wounded soldier they receive. But this sum does not cover all expenses, quite apart from the fact that hospitals have their duty to civil patients as well. By building hut wards and emergency structures of various kinds many institutions are still able to take in as many ordinary patients as before. All the hospitals continue to receive a large proportion of them, and the awakening of the national conscience which has led to the above offer of a Government grant for wounded soldiers must penetrate every civilian also, and lead him "to do his bit," as we say, by subscribing to the institutions in whose good keeping the lives of so many of our friends, sons, brothers, and husbands are confided to-day.

The part which the clergy have taken in bringing home to the people the need for prosecuting the war and the necessity for personal service has been one of the most remarkable features of the past few months. Almost every pulpit has echoed the call to arms. Every pulpit certainly has preached the gospel of personal service. Let the clergy, then, be reminded with gratitude and pride that Hospital Sunday was founded by one of their own order, Canon Miller, in 1859. Its gospel of personal service is their gospel, its protagonist one of themselves. Indeed, it may be said with truth that Canon Miller founded Hospital Sunday, not only as a means for organising the Central collection on behalf of the voluntary hospitals, but because the great thought came to him that the voluntary system itself was but the doctrine of personal service organised and put into practice with reference to the healing of disease. Hospitals, of course, have paid officials; but they depend almost exclusively for their management and control on the brains and time of men and women who voluntarily give their service on committees, as chairmen, treasurers, in ladies' guilds, and subsidiary organisations of all sorts. The medical staffs, as we all know, are not, as a whole, paid anything for their services, but take their material reward in the experience they gain and the prestige which is inherent in their positions. The paid officials are brought up equally in the same tradition. According to commercial standards, beyond a doubt, they are much underpaid, and gain their reward in the satisfaction which comes to those who, means for bed and board once granted, devote themselves to superselfish work. Thus, in short, hospital work is personal service given by all, paid and unpaid, who engage in it, and Hospital Sunday, on which this gospel of service is preached, may be regarded as the festival of Churchmanship actually at work in the world. Founded by the clergy, carried on by them, and this year with the message it has taught embraced by the whole nation, what preacher will not thrill with pride as he repeats the claim of this glorious gospel, and what congregation will be so dull to its meaning as not to give of their best, that the sum total of hard cash subscribed may be as much a "record" this year as the work of the hospitals has been?

### HOSPITALS AND THEIR SPECIAL NEEDS.

**Acton Hospital, Gunnersbury Lane, Acton.**—During the whole of the present year this hospital has been exceedingly full, and the calls on it have been greater than in any previous year. On the other hand, the subscriptions and donations have shown a falling-off during the last eight months, and it has been necessary to use the small reserve to a considerable extent.

**Bolingbroke Hospital (Incorporated), Wandsworth Common, S.W.**—Funds are urgently needed to meet the increased cost of maintenance. Nearly half a million people living in South-West London greatly depend on this hospital in time of sickness or accident.

**British Hospital for Mothers and Babies, Woolwich.**—The hospital is urgently in need of rebuilding, and a three-acre freehold site has been acquired for this purpose. Funds in hand amount to £11,500, but at least double that sum is required. At present patients have frequently to be refused admittance. Many of them are the wives of sailors, soldiers, and workers at the Arsenal, and the number of extra workers at the Arsenal who have settled in Woolwich for the war has accentuated the lack of accommodation at the hospital. The nurses' quarters are uncomfortable, the foundation of an ante-natal clinic is postponed, and training possibilities are greatly curtailed through lack of space.

**Canning Town Women's Settlement Hospital, Balaam Street, Plaistow, E.**—With the larger hospitals crowded, additional responsibilities are thrown upon the smaller ones. The sick poor to whom this hospital ministers in Canning Town have more claim upon the giving public now than they have ever had.

**Central London Throat, Nose, and Ear Hospital, Gray's Inn Road.**—The work of this hospital has not been interrupted during the past or present year. Eight beds were taken over by the War Office, and many wounded have been treated. Soldiers from the regular Army and recruits have been sent in large numbers by the medical officers of their respective corps. The wives and children of soldiers and Belgian refugees have been treated free of cost. Ten members of the staff are on active service, and ladies have been trained in surgical nursing at the request of the Red Cross authorities. Funds are diminished, and help is asked.

**Charing Cross Hospital, Agar Street, Strand, W.C.** Last year the hospital was enlarged from a basis of 150 to 250 beds, through the reopening of the wards closed so long for lack of funds. But this year there is also a heavy programme to be faced. Repairs, external and internal, renewal of the whole engineering plant, and the annual cleaning have to be undertaken. These at the least will cost £5,000. Every penny is spent economically, but it is essential that the hospital should be kept open on its new basis and in the utmost state of efficiency. One hundred beds were set aside by the hospital, and over 600 wounded soldiers have been treated. Special diet has been provided for these, and the whole cost for treating them has been borne by the hospital. Money is needed for this work, and flowers, fruit, provisions, books, etc., would also be welcome. Thanks to generous friends who have lent their houses, it has been possible to find convalescent accommodation for the wounded, and at the same time run the civilian Home to its full extent. At present forty-five soldiers are being provided for, and fifty civilians of both sexes. The Council feel sure that, judging from the generous response they have already had, they will get the support now asked for to carry them on, both at the hospital and in the Home.

**Chelsea Hospital for Women, Fulham Road, S.W.**—It is estimated that the sum of about £18,000 is still required to enable the committee to complete the rebuilding fund. Special facilities are being given for the treatment of wives and other near relatives of soldiers at the Front. Many Belgian refugees are also being gratuitously treated. Contributions to these objects and towards the maintenance of the hospital and its convalescent home are very greatly needed.

**Cheyne Hospital for Sick and Incurable Children, Chelsea.**—Both the hospital and its country branch at St. Nicholas-at-Wade, Kent, continue quite full, but the income is about £600 less than the expenditure, and additional help is badly needed.

**Children's Hospital for the Treatment of Hip Disease, Sevenoaks.**—The year closed with a deficit of £88. This was due mainly to the falling off of donations owing to the war, and the increase in the expenditure for foodstuff. There is therefore special need for increased help during the present year.

**City of London Hospital for Diseases of the Chest (Victoria Park, E.).**—A scheme of improvements, comprising new passenger lift and additional balcony accommodation (as recommended by King Edward's Hospital Fund), the installation of electric current, combined with a rearrangement of the boiler system, and extension of special departments, together with new sanitary blocks, had to some extent been embarked upon just before the war, but has now unfortunately been curtailed owing to the anxious financial situation. The falling-off of contributions, largely due to the impossibility of holding the usual annual dinner in November last, has made the demand on an accumulated balance of income (resulting from certain exceptional legacies) set aside for the improvements a serious factor in the financial position. Twenty beds have been provided for soldiers invalided from the British and Allied Forces, and support towards their maintenance is urgently needed. Although the committee recognise the need of exceptional efforts to meet the grave requirements of a special character in connection with the war, they feel that they cannot too strongly asseverate the importance of continued adequate provision of treatment of the sick amongst the civil population—which include a large number of the wives and children of our sailors and soldiers—especially as distressing conditions such as the present tend to increase the need. The committee hope that all those who have assisted the hospital in the past will not fail to support it in the present difficult circumstances, and trust that its value and needs will not be overlooked by the public generally.

**City of London Lying-in Hospital.**—This hospital is still suffering from the effects of the heavy debt which was incurred when rebuilding in 1906. The rebuilding was absolutely essential on account of the construction of a tube railway opposite the hospital, which rendered the old structure unsafe, and it became necessary to borrow the sum of £24,000 from the bankers. After nine years, by the exercise of strict economy and persistent appeals, the loan has been reduced to £4,700, and the greatest difficulty is experienced in reducing it further on account of the financial stringency caused by the war. At the present time the work of a lying-in hospital is particularly important, since its labours are constructive and tend to repair to some slight extent the immense damage done to the nation by the killing of the flower of its young manhood. This hospital not only lavishes every care upon thousands of poor mothers whose infants would otherwise be born in surroundings of dirt and neglect, but it now welcomes without any formality the wives of our sailors and soldiers.

**East End Mothers' Lying-in Home, Commercial Road, E.**—To carry on the work of the home new subscriptions and two bicycles for out-patients' midwives are wanted. There are fifteen new bedsteads for the use of sailors' and soldiers' wives.

**East Ham Hospital, Shrewsbury Road, E.**—During the past year this hospital has been extended, but the annual house-to-house collection has fallen off considerably, and the annual carnival, which produces about £250, cannot be held; these are serious considerations to a hospital supported entirely by voluntary contributions. Ten beds have been given up to the wounded, and these have been fully occupied since October.

**East London Hospital for Children, Shadwell, E.**—Reduced incomes and money diverted to war funds have materially affected the funds of this hospital, which is at present unavoidably living on borrowed money, and, unless an unexpected windfall happens, will be obliged to continue to borrow until its security is exhausted. The board propose to hand over the convalescent home at Bognor for the use of the wounded.

**Epsom and Ewell Cottage Hospital, Surrey.**—Last year the deficit in ordinary income and expenditure was £83. Some eighty cases from the Public Schools Battalions of the Royal Fusiliers quartered at Epsom have been treated.

**Evelina Hospital for Children, Southwark Bridge Road, S.E.**—Owing to its situation, the "Evelina" is rarely seen by wealthy people, and suffers accordingly, although it is the only large children's hospital for the whole of South London, and is situated in its most destitute district. Although obviously nothing can be done for the wounded at the "Evelina," as its arrangements are such that it can only be used as a hospital for children, many of its patients are the little ones of our brave sailors and soldiers who are fighting for their country. The Evelina Hospital is doing exceedingly good work, as may be gathered from the fact that over 1,000 in-patients and about 55,000 out-patients are dealt with each year. The annual cost of maintenance is £8,000, but unfortunately the hospital does not gain the support it merits, and the committee find it necessary to make a strong appeal for new annual subscriptions, which are urgently required; at the close of 1914 there was a deficit of £650; in times like the present this is disastrous.

**Eversfield Chest Hospital, West Hill Road, St. Leonards-on-Sea.**—£2,000 is required to continue without delay the work of erecting a cliff wall to prevent the serious crumbling of the cliff which bounds the grounds on the south side of the hospital. Larger sitting rooms for men and women patients, a larger dining hall, and better accommodation for nurses are also needed. The smallest donations will be welcomed. Frequent applications have been made by the authorities for beds for tuberculous sailors and soldiers discharged from the Services, which could be entertained if means were forthcoming to erect a temporary building for them.

**French Hospital and Dispensary, Shaftesbury Avenue.**—This hospital is open to all foreigners speaking French, and in 1914 898 patients were admitted. The hospital placed twenty-five beds at the disposal of the British War Office and Admiralty, and the French Convalescent Home at Brighton also offered thirty beds. Over 100 wounded (Belgian and English) have been received, and ten Belgian refugee ladies were also given shelter at the Convalescent Home.

**Great Northern Central Hospital, Holloway Road.**—This is the largest general hospital in North London, and ministers to a population of over half-a-million people, who, being largely of the working classes, are dependent upon its assistance in times of sickness or accident. In normal times the hospital has 211 beds in London and Clacton, and 2,600 in-patients and 22,000 out-patients are treated annually at a cost of about £21,000 a year, the greater part of which has to be obtained from voluntary sources. Among the most pressing needs of the hospital, apart from funds required for general maintenance, are contributions towards building an up-to-date Nurses' Home, which it is estimated will cost more than £12,000; towards this amount about £1,400 has been collected. In the present national crisis the committee have placed 130 beds at the disposal of the military authorities for sick and wounded soldiers, and to meet this emergency a large number of additional beds have been provided. Owing to the increased accommodation and to the extra work now being done additional expenditure is entailed, and funds are more than ever needed to meet current accounts. Already some 450 soldiers have passed through the wards. There is always a long list of patients awaiting admission. Contributions in money or in kind are earnestly solicited, and would be gratefully acknowledged by the Secretary, Mr. Gilbert G. Panter.

**Grosvenor Hospital for Women, Vincent Square, Westminster.**—Funds are needed to carry on the work of this hospital. Six beds have been placed at the disposal of the War Office for sisters of the Territorial Field Force.

**Guy's Hospital, S.E.**—The work of the hospital has been materially increased, and its efficiency maintained unimpaired during this time of strain. 115,982 out-patients and 9,924 in-patients were treated in 1914. The ordinary expenditure amounts to £76,237, and the income from endowments is £48,264. For the large difference between assured income and ordinary outgoings, and for the extraordinary expenditure necessary from time to time, the hospital is dependent on voluntary support, and the ordinary income still falls

[*Continued on p.* 16.

### Nearly Two Million Sufferers Helped by the Hospitals.

### A SINGLE YEAR'S ROLL-CALL OF THE SICK.

In the last year for which complete figures are available, the immense total of *one million nine hundred and fifty-five thousand one hundred and twenty* patients were treated at the voluntary hospitals and dispensaries of London, the endowed hospitals of St. Bartholomew's, Guy's, and St. Thomas's, and the infectious hospitals of the Metropolitan Asylums Board. These figures only include the in-patient cases treated to a termination in the wards of the hospitals and the number of new out-patient cases treated in the out-patient departments and dispensaries, and may be taken as showing as nearly as possible the number of separate cases dealt with in the hospitals and dispensaries of the Metropolis. This is over three hundred thousand less than were treated in the previous year, three hundred and ten thousand less having attended the out-patient departments and dispensaries against a slight increase of seven thousand in the in-patients.

861,416. Surgical Patients.

**Patients Suffering from Surgical Diseases.**—Of the whole number of patients received by the hospitals, *eight hundred and sixty-one thousand four hundred and sixteen* required surgical treatment in addition to those treated in the special departments and in the hospitals for diseases of the eye, nose, throat, and ear. "Surgical" diseases include not only all accidents such as broken bones, fractured skulls, mangled limbs, and all manner of displacements and crushings of sensitive parts and organs, but also abscesses, ulcerations, cancers, and tumours of all kinds; in short, all those injuries which may be produced by accident or pathological process, and which may be dealt with either by hand or instrument. It is not easy to realise that, including the special departments of our large institutions and the special hospitals, about one million one hundred and twenty-five thousand patients are treated annually in the London hospitals for diseases requiring surgical treatment.

619,829. Medical Patients.

**Patients Suffering from Medical Diseases.** — *Six hundred and nineteen thousand eight hundred and twenty-nine* persons received medical treatment. By medical diseases are meant those diseases which are situated either as to their source and origin or in their entirety in one or the other of the three great cavities of the body. They include rheumatic fever, pneumonia, pleurisy, bronchitis, diseases of the stomach, bowels, liver, kidney, bladder, and pancreas, every kind of heart disease, many forms of brain injury, dyspepsia, constipation, most nervous diseases, and other ailments, many of them serious and many of them dangerous to life, or at least to the useful existence of the individual. Most of these diseases are out of sight; the diagnosis of their nature and extent, and the successful treatment of them, is dependent on the doctor's scientific knowledge. Remembering this, try to realise that in the hospitals of London over six hundred thousand persons received treatment for these diseases at the hands of the foremost physicians of the day, free of cost to the patients themselves.

166,553. Eye.

**Patients Suffering from Eye Affections.**—*One hundred and sixty-six thousand five hundred and fifty-three* persons were treated in the special departments of the general hospitals or by the ophthalmic hospitals of London. It is certain that very many of these cases must have entailed terrible suffering, and many doubtless would have terminated in total loss of sight but for the skilful treatment they have received at the hospitals. Who can say how many have been saved from becoming practically helpless in the world?

### THE ROLL-CALL OF THE SICK.—*continued.*

**Patients Treated at Special Hospitals for Children.**—Included in the patients mentioned at the commencement of this article are *one hundred and seventy-seven thousand one hundred and nineteen* children who were sent from homes where they could not be properly attended to for treatment in the special hospitals for the little ones.

**177,119. Children.**

**Patients Suffering from Diseases of the Ear, Nose, and Throat.**—At the special hospitals or special departments devoted to these diseases *ninety-seven thousand and sixty-three* were treated. The affections and diseases of these organs, which are intimately connected, involve temporary and often permanent impairment of hearing, swallowing, and breathing. These functions are performed with so little effort on our part that, unless experience has taught us, it is difficult to understand what it would mean to us if we suddenly had to suffer from one or other of these affections.

**97,063. Ear and Throat.**

**Diseases of Women and Motherhood.**—*Eighty-six thousand and thirty-three* women were treated at the Metropolitan voluntary hospitals for those diseases which are peculiar to their sex. Here it is not only our sympathy which is appealed to, but our patriotism as well. Here there is an actual demand for the payment of a debt we most justly owe. The very heart and strength of the nation lies in the home life, and the soul of the home life is the woman—the mother.

**86,033. Women.**

**Patients Suffering from Diseases of the Skin.**—During the year *fifty-six thousand two hundred and eighty-nine* persons were treated for skin diseases in London. It is, perhaps, more difficult to bring home to people the claims which sufferers from skin diseases have upon their sympathy than it is in any of the other diseases which we are considering. There is not, here, as a rule, the pain, nor the danger to life, nor even such risk of permanent disablement as is the case with many of the others; but let us remember what the result would be were there no hospitals for the sufferers to go to.

**56,289. Skin.**

**Patients Suffering from Consumption.**—*Thirty-one thousand three hundred and fifty-two* patients suffering from phthisis or consumption were treated at the hospitals of London during the year. The very word "consumption" makes us afraid. There are few of us who have not seen something of its ravages, of its cruelty. Truly may consumption be called the curse of our climate. It respects neither persons nor estate, neither rich nor poor, old or young.

**31,352. Consumption.**

**Patients Suffering from Fever.**—The number of persons who were treated for the class of fevers which are usually removed to a fever hospital was *twenty-six thousand four hundred and forty-seven.* This figure is, however, a misleading one, because the term fever includes much besides this class of fever. Measles, for instance, prevails in London to such an extent that more deaths occur from it than from scarlet fever. The excellent service rendered by the London Fever Hospital entitles it to the gratitude of all householders.

**26,447. Fever.**

**Patients Suffering from Paralysis and Epilepsy.**—*Ten thousand one hundred and thirty-eight* stricken with paralysis, epilepsy, and kindred ailments received treatment at the general hospitals and at hospitals devoted to these maladies. To workers busy with hand and brain these sufferers must particularly appeal. It is impossible to dissociate nervous breakdown from the toil and hurry of existence, especially in a vast centre like London. It is appalling to think that at any moment any one of us may be struck down, perhaps without the slightest warning. No disease is more sudden than paralysis, surely none more pitiful.

**10,138. Paralysis.**

So this great army of sufferers, numbering nearly two millions, claims our sympathy and our help year by year. To the strong, to those in health who are able to provide for those dependent upon them, to those who know what ill-health means, who have suffered from disease of one kind or another, and who, either in the hospital or under the skill and care of the doctors and nurses trained in the hospitals, have been restored to health and usefulness, we confidently appeal on behalf of the London hospitals.

### THE ROLL-CALL OF THE SICK.

| | | | |
|---|---|---|---|
| Sufferers needing Surgical Aid | 861,416 | Sufferers from Skin Diseases | 56,289 |
| Sufferers needing Medical Care | 619,829 | Consumptives | 31,352 |
| Sufferers from Eye Troubles | 166,553 | Fever Patients | 26,447 |
| Diseases of the Ear, Nose, and Throat | 97,063 | Paralysis and Epilepsy | 10,138 |
| Diseases of Women | 86,033 | Total | 1,955,120 |

### Hospitals and their Special Needs. [Continued from p. 13.

short of the ordinary expenditure by over £6,000. The Governors urgently plead for additional donations and legacies in order to lessen the building debt, to endow the medical school, which practically depends upon students' fees, and for the medical school research fund. In view of the extreme pressure on the available beds of the hospital on the part of the sick poor of the civil population, the Governors did not feel justified in reserving large numbers of beds for wounded, but they offered to provide and erect in the hospital "Park" huts to accommodate 150 beds, and to equip and work them free of cost. The Governors also earnestly appeal for new annual subscriptions and donations to provide for the deficiency between income and ordinary outgoings to enable them to meet the expenses incurred by the double demand of caring for the sick poor of the civil population and the treatment of military patients, to whom a special ward of forty-eight beds, recently renovated and laid with rubber flooring, has been entirely devoted. In addition, a considerable number of men have received treatment for minor surgical ailments to enable them to enlist, and a further ward has been set apart for this purpose.

**Hampstead General and North-West London Hospital.**—The amalgamation of these two hospitals was effected in 1908 through the instrumentality of King Edward's Hospital Fund, and 1,520 patients were admitted to the hospital during the past year, while the attendances of the out-patients' department averaged over 2,000 weekly. But the development of the hospital in the special circumstances indicated has been too rapid to allow of the income being correspondingly augmented, and the total indebtedness of the institution is now more than £7,000. The committee consequently make an earnest appeal for new annual subscriptions and donations.

**Hospital for Consumption, Brompton, S.W.**—For seventy-five years this has been the leading London hospital for chest troubles. It has a sanatorium and convalescent home in Surrey, and in the combined institutions about five hundred beds are constantly occupied. Many British sailors and soldiers, as well as French and Belgian soldiers, have been admitted to the Brompton Hospital, and also to the Sanatorium and Convalescent Home at Frimley since the outbreak of the war. Special arrangements have also been made for the admission of dependants of sailors and soldiers serving with H.M. Forces if needing treatment. Money is the one essential required, and the committee appeal to the generous and patriotic public to come to their aid with funds sufficient to carry on the work.

**Hospital for Diseases of the Throat (with which is amalgamated the London Throat Hospital, Great Portland Street, W.), Golden Square, W.** This hospital is feeling severely the need for support this year. Thirty beds have been reserved for wounded, and 139 such cases have been treated. Besides this, many recruits as well as the dependants of men serving in H.M's Forces and those of our Allies have been given free treatment both as in- and out-patients.

**Hospital for Sick Children, Great Ormond Street, W.C.**—It is anticipated that unless the unexpected happens during the year 1915, a deficit between assured income and expenditure of about £3,000 will have to be met, and the committee earnestly appeal for the work of this hospital, lest it should be overlooked at a time when the "grown-ups" are, perhaps inevitably, especially attracting the attention of the generous public. In view, therefore, of the precarious times through which the hospital is passing, the committee most sincerely beg for the continuation of the support of the generous public to enable them to maintain the highest standard of usefulness in their treatment of the sick and suffering children of the poor.

**Hospital for Women, Soho Square, W.**—During 1914 1,083 patients were admitted, a record for the hospital, while there were usually about 100 patients on the waiting list. New annual subscribers are wanted to help to reduce the mortgage debt, which now stands at £4,500, and costs the hospital £192 10s. a year for interest alone.

**Infants' Hospital, Vincent Square, Westminster, S.W.**—This hospital is at the present time £700 in debt on account of expenses of maintenance, and, after reckoning all the money in prospect—chiefly promised subscriptions—there remains a further sum of about £1,700 to be raised to meet the expenses of maintenance for this year. The hospital has at present no expenses in connection with the care of wounded sailors and soldiers, but accommodation has been offered by the hospital to the War Office, although the offer has not yet been accepted.

**Kensington and Fulham General Hospital.**—Five members of the medical staff, the secretary, and the matron are on Service. The hospital's honorary staff have offered their professional services to meet local requirements, whilst ex-Service men and would-be recruits are being treated gratuitously by the hospital. Funds are urgently needed to enable the work of the hospital to proceed.

**King Edward Memorial Hospital, Ealing.**—Large sums have been subscribed locally to the two temporary military hospitals at Southall and Ealing, and this must necessarily affect to some extent the financial position of this hospital. Arrangements were made to treat ten wounded soldiers free, and three months ago twenty additional beds were reserved for the same purpose, to be paid for at the regulation amount per diem. These are all in use, but it has meant the additional expenditure up to December 31, 1914, of £125. Therefore voluntary help is much needed at the present time.

**King's College Hospital, Denmark Hill, S.E.**—This is one of the newest of London's hospitals. The removal of the hospital from the Strand district to South London has unfortunately had a serious effect on its finances. It has necessarily lost its local connection, and is only gradually building up a new one in South London. It is hoped, however, that residents in this district will liberally respond, despite the exceptional calls made at the present time, for the hospital is in urgent need of support for its ordinary work, and is still heavily burdened by a large debt on the new buildings, which have now all been taken into use. On the outbreak of the war a considerable portion of the hospital was placed at the disposal of the War Office for the use of the 4th London General Hospital, to which many of the members of the Honorary Medical Staff are attached, retaining the casualty department, and at least four wards for the use of the civilian population. The buildings are well adapted for the purpose, and it has been found possible to accommodate upwards of 700 patients, together with the increased staff which these numbers entail. It is urgently hoped that the work may not be crippled in any way through lack of funds.

**Kingston-on-Thames Victoria Hospital.**—Though at present comparatively unaffected by the war, for the beds offered to the War Office are as yet unoccupied, the maintenance of the regular subscription list is the great essential and the great difficulty of this hospital.

**London Homœopathic Hospital, Great Ormond Street, W.C.**—To provide the sum to be raised by December 31 of the current year to pay off debts to capital, £14,844, unavoidably incurred in the maintenance of the hospital, many generous donations are necessary, and it is hoped that substantial help will be forthcoming before the year closes.

**London Hospital, Whitechapel, E.**—The work of the London Hospital continues to increase, and during the past year a greater number of patients have been treated there than in any previous year. Despite the requirements of the war, by slightly rearranging the wards the number of beds available for civilians has not been seriously curtailed. By reducing research work and limiting the functions of the hospital to the actual treatment of patients, many doctors and nurses have been enabled to join the Forces. At the same time, while all schemes involving outlay of capital have as far as possible been postponed and the hospital has retrenched its expenditure in various ways, funds are urgently needed to carry on the work. In 1914, 18,300 ordinary in-patients were treated alone, and since the war there has been a general rise in the price of all commodities: drugs and chemicals were, in particular, affected, certain of German origin were unobtainable. Voluntary contributions, moreover, were being directed from the hospital to the support of charities of a more directly patriotic nature. In addition to this, the London Hospital placed 500 beds at the disposal of the naval and military authorities, and was actually the first hospital in London to receive wounded soldiers. Up to December 31, 1914, 1,481 wounded had been treated. To meet the demand for nurses a large number of paying probationers have been received, and great numbers of orderlies have been trained.

**London Lock Hospital.**—This hospital, which is absolutely unendowed and in great need, devotes all its energies, and has done so since its foundation in 1746, to the treatment of venereal diseases. One of its principal and most successful objects is to eradicate these diseases from the children for the benefit of the coming generation. It has the most pathetic children's hospital ward in the kingdom, which must strongly appeal to everyone. Help is urgently needed to enable the institution to clear off a deficit of over £5,000, and the committee make an earnest appeal for assistance. The committee have just issued a special emergency appeal for funds, and the hospital is treating both English and Belgian soldiers; while at the present time there is in their female hospital a young Belgian refugee, a victim of the rapine and pillage of the German invasion.

**Margaret Street Hospital for Consumption, 26 Margaret Street, W., and Fairlight Sanatorium, Hastings.**—Funds are urgently needed to complete the rebuilding of the hospital now almost completed, and at Fairlight for a new wing to accommodate the nursing staff, who are at present very inadequately housed and have but one small room for rest, recreation, reception of visitors, and meals. A bed has been given up for a consumptive Belgian refugee, who was received free of all charge.

**Metropolitan Ear, Nose, and Throat Hospital, Fitzroy Square, W.**—When the war broke out this hospital was placed at the disposal of the naval and military authorities. Beds were set apart, and have been continuously occupied by the wounded, without preventing adequate treatment for civilian patients. In addition to the wounded in the wards, a large number of soldiers have been treated in the out-patient department, not only from the Front, but also from amongst those under training to go to the Front. The additional work occasioned by the war has been a great strain on the finances of the hospital.

**Metropolitan Hospital, Kingsland Road, N.E.**—The most recent events in the war have confirmed the Committee in the wisdom of making ample provision for the civilian population during its progress, having regard to the circumstances of the district and its population. Attached to the hospital there will shortly be opened the adjacent L.C.C. Enfield Road School, for the accommodation of 202 sick and wounded soldiers. The two top wards, at present used for housing nurses, are about to be opened for thirty-six more soldiers; so that in all about 270 soldiers will be treated as in-patients at the Metropolitan Hospital. It is urgently hoped that all friends and supporters of this excellent and thoroughly deserving charity will not only lend their personal aid, but will urge their friends to do so. The maintenance fund to date is far behind the usual figure; but it is hoped that other supporters and friends of the hospital who know, and therefore value, its work will follow the example of the Committee, its medical staff, and practitioners in its neighbourhood, and afford it all the assistance in the present emergency that may be in their power. For those who cannot send money, gifts of clothing, food, fruit, eggs, etc., and flowers are very acceptable, and should be addressed to Mr. J. Courtney Buchanan, Secretary and House Governor, Metropolitan Hospital, Kingsland Road, N.E. Cheques should be crossed "Metropolitan Hospital, a/c Glyn, Mills, Currie & Company."

**Middlesex Hospital, W.**—During the year 1,570 in-patients received treatment, while the total number of attendances in the out-patient department was 33,460. In the Cancer Charity 169 patients were

[*Continued on p.* 20.

### 1914.

### A Year's Work in the Hospitals and Medical Charities of London.

### ST. MARYLEBONE AND WEST CENTRAL DISTRICT.

Comprising St. Marylebone, St. John's Wood, Bloomsbury, Holborn, &c.

| No. of Beds. | No. of Beds Daily Occupied. | Hospitals. | In-patients. | Out-patient Attendances. | Total Expenditure. | Income. | | | Total Income. | Legacies not included in preceding column. |
|---|---|---|---|---|---|---|---|---|---|---|
| | | | | | | Charitable. | Proprietary. | Patients' Payments. | | |
| | | | | | £ | £ | £ | £ | £ | £ |
| 74 | 48 | French | 898 | 17,897 | 6,030 | 5,152 | 1,522 | ... | 6,674 | 1,441 |
| 50 | 45 | Italian | 812 | 7,843 | 3,891 | 2,301 | 1,244 | ... | 3,545 | 180 |
| 164 | 131 | London Homœopathic | 1,562 | 59,510 | 14,830 | 4,830 | 3,585 | 2,368 | 10,783 | 5,050 |
| 119 | 100 | SS. John and Elizabeth | 873 | ... | 10,424 | 1,634 | 7,089 | 1,642 | 10,365 | ... |
| 445 | 382 | The Middlesex | 5,908 | 154,978 | 51,028 | 18,379 | 10,838 | ... | 29,217 | 32,522 |
| 104 | 93 | Alexandra, for Children | 103 | 1,490 | 5,400 | 3,639 | 855 | 877 | 5,371 | 500 |
| 240 | 211 | Hospital for Sick Children | 3,030 | 81,456 | 23,873 | 12,540 | 6,260 | 241 | 19,041 | 7,594 |
| 24 | 17 | S. Monica's, for Children | 118 | ... | 1,459 | 466 | 222 | 209 | 897 | 250 |
| 82 | 68 | Queen Charlotte's Lying-In | 1,904 | 20,680 | 8,578 | 4,698 | 981 | 1,016 | 6,695 | ... |
| 66 | 59 | New Hospital for Women | 1,194 | 33,650 | 8,251 | 2,946 | 1,239 | 2,200 | 6,385 | 5 |
| 67 | 54 | Samaritan Free | 934 | 14,823 | 7,127 | 4,620 | 469 | ... | 5,089 | 1,235 |
| 200 | 178 | National for the Paralysed, &c. | 1,101 | 44,303 | 19,109 | 8,248 | 2,977 | 4,828 | 16,053 | 6,048 |
| 70 | 70 | Hospital for Epilepsy, &c. | 397 | 18,890 | 5,657 | 3,603 | 211 | 1,334 | 5,148 | 450 |
| 52 | 48 | West End, for Epilepsy, &c. | 254 | 31,016 | 6,346 | 4,517 | 1,388 | 991 | 6,896 | 270 |
| 28 | 18 | Central London Ophthalmic | 367 | 27,387 | 2,591 | 2,281 | 85 | ... | 2,366 | ... |
| 16 | 15 | Western Ophthalmic | 384 | 26,212 | 2,127 | 2,038 | 128 | 388 | 2,554 | 50 |
| 180 | 144 | Royal National Orthopædic | 1,097 | 35,028 | 11,793 | 8,389 | 1,397 | 1,681 | 11,467 | 6,138 |
| 38 | 31 | Florence Nightingale Home | 458 | ... | 4,875 | 1,832 | 297 | 2,866 | 4,995 | 50 |
| ... | ... | National Dental | ... | 26,526 | 2,362 | 1,045 | ... | 1,274 | 2,319 | ... |
| 14 | 9 | London Throat | 471 | 11,861 | 1,650 | 644 | ... | 857 | 1,501 | ... |
| 49 | 44 | The Middlesex Cancer | 199 | 946 | 5,679 | 1,647 | 2,581 | ... | 4,228 | 854 |
| 20 | 10 | Metropolitan Ear, &c. | 441 | 10,185 | 1,671 | 753 | 33 | 664 | 1,450 | ... |
| 2,102 | 1,775 | | 22,505 | 624,681 | 204,751 | 96,302 | 43,301 | 23,436 | 163,039 | 62,637 |
| | | Dispensaries. | | | | | | | | |
| ... | ... | London Medical Mission | ... | 31,093 | 1,820 | 606 | 463 | 107 | 1,176 | 2,100 |
| ... | ... | Margaret Street, for Consumption | ... | 8,873 | 738 | 530 | 69 | ... | 599 | ... |
| ... | ... | St. John's Wood Provident | ... | 12,570 | 584 | 280 | 28 | 198 | 506 | ... |
| ... | ... | St. Marylebone General | ... | 16,595 | 1,202 | 528 | 202 | 164 | 894 | 50 |
| ... | ... | Western General | ... | 23,327 | 960 | 620 | 113 | 24 | 757 | 250 |
| 2,102 | 1,775 | | 22,505 | 717,139 | 210,055 | 98,866 | 44,176 | 23,929 | 166,971 | 65,037 |

### WESTMINSTER DISTRICT.—Comprising Westminster City and Liberties.

| No. of Beds. | No. of Beds Daily Occupied. | Hospitals. | In-patients. | Out-patient Attendances. | Total Expenditure. | Charitable. | Proprietary. | Patients' Payments. | Total Income. | Legacies not included in preceding column. |
|---|---|---|---|---|---|---|---|---|---|---|
| | | | | | £ | £ | £ | £ | £ | £ |
| 250 | 146 | Charing Cross | 2,568 | 77,122 | 21,488 | 19,073 | 4,142 | 181 | 23,396 | 4,493 |
| 213 | 174 | Westminster | 2,480 | 62,554 | 21,879 | 7,056 | 5,126 | ... | 12,182 | 6,502 |
| 163 | 147 | Ventnor, for Consumption | 487 | ... | 13,956 | 7,086 | 3,363 | 7,113 | 17,562 | 6,180 |
| 37 | 32 | Grosvenor, for Women & Children | 445 | 6,922 | 2,943 | 1,613 | 415 | 840 | 2,868 | 245 |
| 67 | 67 | Hospital for Women | 1,083 | 14,833 | 7,408 | 4,808 | 621 | 1,256 | 6,685 | 997 |
| 25 | 21 | Gordon, for Fistula | 318 | 3,232 | 2,692 | 438 | ... | 1,665 | 2,103 | 135 |
| 24 | 20 | National, for Diseases of Heart | 175 | 18,362 | 3,473 | 1,957 | 348 | 772 | 3,077 | 432 |
| 40 | 32 | Royal Westminster Ophthalmic | 927 | 38,563 | 3,632 | 2,075 | 1,329 | ... | 3,404 | 950 |
| 20 | 13 | Royal Ear | 542 | 11,340 | 2,249 | 1,015 | 5 | 706 | 1,726 | 1,350 |
| ... | ... | Royal Dental | ... | 55,104 | 5,242 | 5,729 | 1,611 | ... | 7,340 | 1,225 |
| 32 | 29 | St. Peter's, for Stone | 452 | 36,685 | 4,860 | 1,640 | 581 | 2,418 | 4,639 | 54 |
| 50 | 38 | Infants' Hospital, Vincent Square | 365 | 3,828 | 3,114 | 2,924 | 78 | ... | 3,002 | ... |
| 40 | 32 | St. John's, for Skin Diseases | 278 | 37,175 | 5,104 | 4,342 | 50 | 1,919 | 6,311 | ... |
| 57 | 28 | Throat Hospital, Golden Square | 983 | 44,937 | 5,801 | 2,219 | 45 | 4,134 | 6,398 | 1,000 |
| 1,018 | 779 | | 11,103 | 410,657 | 103,841 | 61,975 | 17,714 | 21,004 | 100,693 | 23,563 |
| | | Dispensaries. | | | | | | | | |
| ... | ... | Public | ... | 9,161 | 658 | 281 | 220 | ... | 501 | 108 |
| ... | ... | St. George's, Hanover Square | ... | 2,650 | 396 | 306 | 40 | 97 | 443 | ... |
| ... | ... | Western | ... | 16,470 | 1,324 | 554 | 512 | 680 | 1,746 | ... |
| ... | ... | Westminster General | ... | 11,727 | 686 | 352 | 231 | 78 | 661 | ... |
| 1,018 | 779 | | 11,103 | 450,665 | 106,905 | 63,468 | 18,717 | 21,859 | 104,044 | 23,671 |

### CITY AND EAST CENTRAL DISTRICT.

Comprising the City, St. Luke's, Shoreditch, Finsbury, and Clerkenwell.

| No. of Beds. | No. of Beds Daily Occupied. | HOSPITALS. | In-patients. | Out-patient Attendances. | Total Expenditure. | Income. | | | Total Income. | Legacies not included in preceding column. |
|---|---|---|---|---|---|---|---|---|---|---|
| | | | | | | Charitable. | Proprietary. | Patients' Payments. | | |
| | | | | | £ | £ | £ | £ | £ | £ |
| 123 | 111 | Metropolitan ... ... ... | 1,788 | 118,885 | 16,566 | 13,378 | 805 | 285 | 14,468 | 4,150 |
| 165 | 146 | Royal Free ... ... ... | 2,396 | 114,492 | 19,458 | 8,272 | 4,019 | 75 | 12,366 | 12,009 |
| 687 | 556 | St. Bartholomew's ... ... | 8,948 | 277,217 | 96,008 | 12,409 | 70,482 | 1,475 | 84,366 | 1,560 |
| 80 | 66 | Royal, for Diseases of the Chest . | 452 | 19,946 | 7,528 | 4,401 | 283 | 122 | 4,806 | 283 |
| 164 | 153 | Queen's, for Children ... ... | 1,736 | 87,582 | 17,081 | 14,160 | 902 | 520 | 15,582 | 3,450 |
| 61 | 48 | City of London Lying-In ... | 1,175 | 9,128 | 6,514 | 1,830 | 4,029 | 404 | 6,263 | 500 |
| 50 | 45 | St. Mark's, for Fistula ... ... | 732 | 6,839 | 5,891 | 4,522 | 1,263 | ... | 5,785 | 1,166 |
| 138 | 106 | Royal London Ophthalmic ... | 2,091 | 121,826 | 13,773 | 9,428 | 1,904 | 507 | 11,839 | 1,413 |
| 30 | 26 | Central London Throat and Ear . | 900 | 48,613 | 4,271 | 1,551 | 323 | 3,036 | 4,910 | 1,104 |
| 1,498 | 1,257 | | 20,218 | 804,528 | 187,090 | 69,951 | 84,010 | 6,424 | 160,385 | 25,635 |
| | | DISPENSARIES. | | | | | | | | |
| ... | ... | Billingsgate Medical Mission ... | ... | 10,066 | 626 | 497 | 7 | 62 | 566 | ... |
| ... | ... | City ... ... ... ... | ... | 15,098 | 874 | 705 | 87 | ... | 792 | ... |
| ... | ... | Farringdon General ... ... | ... | 9,781 | 583 | 317 | 34 | 193 | 544 | ... |
| ... | ... | Finsbury ... ... ... ... | ... | 28,971 | 1,180 | 619 | 194 | 254 | 1,067 | ... |
| ... | ... | Metropolitan ... ... ... | ... | 10,853 | 565 | 251 | 279 | 90 | 620 | ... |
| ... | ... | Royal General ... ... ... | ... | 6,919 | 653 | 213 | 394 | 58 | 665 | ... |
| 1,498 | 1,257 | | 20,218 | 886,216 | 191,571 | 72,553 | 85,005 | 7,081 | 164,639 | 25,635 |

### ISLINGTON AND NORTH-WEST DISTRICT.

Comprising Islington, Holloway, Highbury, Hampstead, Highgate, St. Pancras, Stoke Newington, Tottenham, &c.

| No. of Beds. | No. of Beds Daily Occupied. | HOSPITALS. | In-patients. | Out-patient Attendances. | Total Expenditure. | Income. | | | Total Income. | Legacies not included in preceding column. |
|---|---|---|---|---|---|---|---|---|---|---|
| | | | | | | Charitable. | Proprietary. | Patients' Payments. | | |
| | | | | | £ | £ | £ | £ | £ | £ |
| 209 | 168 | Great Northern Central ... ... | 2,332 | 87,092 | 21,824 | 13,044 | 2,478 | 1,352 | 16,874 | 5,515 |
| 122 | 95 | Hampstead General Hospital ... | 1,342 | 53,542 | 12,817 | 8,353 | 430 | 1,175 | 9,958 | 716 |
| 116 | 84 | London Temperance ... ... | 1,307 | 73,130 | 10,269 | 4,334 | 2,195 | 268 | 6,797 | 1,615 |
| 125 | 114 | Tottenham (Prince of Wales's)... | 1,703 | 90,095 | 11,662 | 10,065 | 237 | 21 | 10,323 | 1,010 |
| 305 | 267 | University College ... ... | 4,578 | 115,312 | 28,510 | 14,595 | 5,699 | 1,107 | 21,401 | 8,683 |
| 110 | 104 | Mount Vernon, for Consumption | 306 | 14,034 | 11,171 | 3,554 | 2,173 | 5,116 | 10,843 | 410 |
| 20 | 19 | Children's Home Hospital, Barnet | 30 | ... | 747 | 636 | 218 | 7 | 861 | ... |
| 160 | 80 | London Fever ... ... ... | 850 | ... | 11,779 | 5,523 | 2,157 | 3,186 | 10,866 | 2,164 |
| 26 | 17 | Invalid Asylum ... ... ... | 188 | ... | 1,130 | 340 | 369 | 226 | 935 | ... |
| 22 | 9 | Bushey Heath Cottage ... ... | 126 | ... | 724 | 383 | 96 | 152 | 631 | ... |
| 25 | 19 | Enfield Cottage ... ... ... | 238 | ... | 1,312 | 1,045 | 110 | 185 | 1,340 | ... |
| 25 | 16 | St. Saviour's Hospital ... ... | 191 | ... | 2,067 | 994 | 75 | 1,104 | 2,173 | 100 |
| 48 | 39 | St. Columba's Hospital ... ... | 127 | ... | 3,890 | 2,888 | 371 | 320 | 3,579 | 455 |
| 25 | 21 | Willesden Cottage ... ... | 353 | 1,803 | 1,472 | 1,232 | 121 | 251 | 1,604 | ... |
| 30 | 19 | Wood Green Cottage ... ... | 273 | 166 | 1,469 | 959 | 82 | 486 | 1,527 | ... |
| 20 | 20 | Santa Claus Home ... ... | 20 | ... | 886 | 786 | 40 | 34 | 860 | 200 |
| 56 | 54 | Hospital for Incurable Children | 17 | ... | 2,141 | 739 | 419 | 984 | 2,142 | ... |
| 18 | 16 | Winifred House, Holloway ... | 26 | ... | 819 | 536 | 66 | 187 | 789 | 10 |
| 18 | 14 | Highgate, All Saints' Home ... | 159 | ... | 466 | 505 | 4 | 3 | 512 | ... |
| 1,480 | 1,175 | | 14,166 | 435,174 | 125,155 | 70,511 | 17,340 | 16,164 | 104,015 | 20,878 |
| | | DISPENSARIES | | | | | | | | |
| ... | ... | Child's Hill Provident ... ... | ... | 3,160 | 128 | 20 | 11 | 97 | 128 | ... |
| ... | ... | Hampstead Provident ... ... | ... | 9,750 | 868 | 208 | 60 | 635 | 903 | ... |
| ... | ... | Islington ... ... ... ... | ... | 43,458 | 729 | 237 | 33 | 541 | 811 | ... |
| ... | ... | Islington Medical Mission ... | ... | 11,370 | 789 | 635 | 124 | 81 | 840 | ... |
| ... | ... | Kentish Town Medical Mission... | ... | 2,900 | 227 | 218 | 4 | 9 | 231 | ... |
| ... | ... | St. Pancras and Northern ... | ... | 9,397 | 693 | 273 | 133 | 237 | 643 | ... |
| ... | ... | Stamford Hill, &c. ... ... | ... | 22,857 | 799 | 530 | 164 | ... | 694 | 22 |
| 1,480 | 1,175 | | 14,166 | 538,066 | 129,388 | 72,632 | 17,869 | 17,764 | 108,265 | 20,900 |

[*For remaining tables see pages* 22 *and* 23.

### Hospitals and their Special Needs.

[Continued from p. 17.

under treatment, and 720 new patients were seen in the electrical and light department. The work of the Cancer Investigation Department continues, and steps have been taken for the establishment of a properly organised Electrocardiograph Department. Owing to the war the staff has been reduced, for many members of the medical and surgical staff have joined the Forces. Three hundred and one wounded soldiers have been treated in the hospital, and no fewer than 1,432 soldiers at the Branch Hospital at Clacton-on-Sea. The Branch Hospital has been increased in its bed capacity and equipped with every appliance required to enable it to deal with the most severe cases. It is one of the three clearing hospitals for the Eastern District. In these circumstances the board trust that the ordinary work of the hospital, as well as the special service which it has undertaken, will not be allowed to suffer for want of adequate support.

**Mildmay Mission Hospital, Bethnal Green.**—Owing to the war all plans and schemes for improvement have been entirely dropped. Many soldiers and many civilians desiring to enlist have been treated in the wards, and thirty beds were placed at the disposal of the War Office.

**Miller General Hospital for S.E. London, Greenwich Road, S.E.**—Contributions are needed towards making good the deficiency in income which will be caused in this year's accounts by the war. The work of the hospital has been carried on, but not without difficulty. Nearly the whole of the in-patient visiting staff are with the Forces, and it is difficult to fill their places. A debt of £4,400 still remains unpaid, and a scheme for providing new out-patients' buildings necessarily remains in abeyance. A ward has been set apart for wounded, and this has been continuously occupied. Moreover, the board are prepared to place the resources of the institution at the disposal of the Government to the extent that may become necessary.

**Mount Vernon Hospital for Consumption, etc., Fitzroy Square, W.**—The committee appeal for funds to complete the extension for women and children at Northwood. This extension will provide 100 additional beds. The foundations are completed, and £5,000 is urgently needed in order that the building may be opened free of debt. These beds are needed especially for children, as the applications exceed the present accommodation. Beds have been reserved free for soldiers suffering from chest diseases contracted at the Front.

**National Dental Hospital (Dental Department of University College Hospital).**—Since the war broke out this special hospital has treated over 4,000 soldiers free, for gas extractions and fillings, besides providing over 100 of them with free dentures. To do this work a panel of over fifty former students was arranged who each gave one or two mornings a week at the hospital for this purpose. Since the War Office undertook to pay for dental treatment for soldiers this panel has been discontinued, but the hospital is still treating a number of cases, especially those which present any difficulties. In doing this work it has been impossible to avoid increased expenditure, and consequently the institution finds itself faced with a present debt of nearly £1,000, and a difficulty in meeting this liability. All this time the staff, as well as the students, have been depleted by absences for military service, leaving increased pressure of work on those who remain. The numbers of ordinary patients have increased, and among these there is a continuous increase in the number who have to be treated quite free, and a decrease in the number who can afford to pay the very small charges asked from those who can afford them, while they are unable to pay a dentist's charges.

**National Hospital for Diseases of the Heart, Westmoreland Street, W.**—This is the only institution in the world solely devoted to the special study and treatment of diseases of the heart. Its special need is £9,000 to repay the debt resulting from compulsory rebuilding in 1913-14. A ward has been placed at the disposal of the War Office, but has not at present been utilised.

**National Hospital for Paralysed and Epileptic, Queen Square, W.C.**—An appeal is made for new annual subscriptions and donations. The hospital has since the commencement of the war received cases of soldiers suffering from nerve injuries and severe mental and nervous shock. Every effort is being made to provide accommodation for the needs of the civil public, and several extra beds have been temporarily added.

**North London or University College Hospital, W.C.** Although the ordinary work of the hospital is proceeding satisfactorily, and the treatment of civilians has not been interfered with, it cannot be denied that the hospital has suffered considerably in the matter of large donations and from the non-payment, at present, of legacies. One hundred beds were given up to wounded soldiers, and these have been fully occupied by British and Belgian wounded. An auxiliary hospital to accommodate 125 wounded soldiers is also being established in the new Eugenics Laboratory at University College. In addition, further treatment has been given to fit men for military service, while 2,000 have been inoculated against enteric. Many of the medical officers and over sixty past and present members of the nursing staff are serving with the Forces.

**Paddington Green Children's Hospital, W.** (Convalescent Home at "Fair View," Slough).—The hospital provides forty-six beds, and there is accommodation at the Convalescent Home for sixteen children in the winter and twenty-four in the summer months. The committee are making a special appeal in aid of their Convalescent Home at Slough. The home is for children who, on leaving the hospital, still need skilled treatment and nursing. The greatest benefit is derived from change of air, and restoration to health is accelerated by a stay at the home. Consequently the usefulness of the hospital itself is proportionately increased by the transfer of patients to this institution. The total expenditure of the hospital during 1914, including a transfer of £530 to the Convalescent Home, amounted to £5,511, and the ordinary income to £4,326, a deficiency of £1,185. This deficit was almost entirely met by the receipt of £400 specially granted for the purpose of reducing the hospital's debt by King Edward's

Hospital Fund, and of £748 from legacies. The amount owing by the hospital to its bankers on December 31, 1914, remained the same as on January 1—viz., £1,100.

**Passmore Edwards District Cottage Hospital, Tilbury, Essex.**—This hospital has also suffered from curtailment of subscriptions and donations in consequence of the war and higher prices of commodities.

**Passmore Edwards Hospital for Willesden, Harlesden Road, N.W.**—There are now 50 beds instead of twenty-five, and funds are much wanted to enable this hospital to treat its soldier and civilian patients.

**Phillips Memorial Homœopathic Hospital and Dispensary, Bromley.**—The increase in the price of food and in gardeners' wages, together with the necessity for having extra nursing and secretarial help, is pressing heavily on the resources of this institution, the work of which has been added to by the reception of wounded soldiers. The one great need is a large increase in annual subscriptions.

**Poplar Hospital for Accidents, East India Dock Road, Poplar, E.**—Funds are needed to enable the hospital to continue to pay its way. Accidents come in at the rate of eighteen per hour every working day, and for sixty years the hospital has been free from debt, but this year food, drugs, and dressings are all dearer. Without withdrawing any of the beds for ordinary patients, twenty beds have been set aside for wounded soldiers, and these have been continuously in use since the middle of December.

**Prince of Wales's General Hospital, Tottenham.**—The hospital needs £11,000 a year, and this amount has to be raised entirely by voluntary contributions. It deals with 30,000 patients every year. An urgent appeal is made for help, as the needs of the vast artisan population of half-a-million, by which the hospital is surrounded, constitute one of the strongest claims upon the wealth of London. The hospital has been accommodating wounded soldiers voluntarily from practically the beginning of the war.

**Queen Charlotte's Lying-in Hospital, Marylebone Road, N.W.**—The hospital is in great need of funds for maintenance. Last year there was a deficiency of £1,497, and this year there has been a falling off in subscriptions, while the considerable increase in the price of food and practically all other requirements has added still further to the difficulties experienced in carrying on the work. Over 700 wives of sailors and soldiers have been treated free of charge, as well as many Belgian and other refugees. Further contributions are earnestly solicited.

**Queen's Hospital for Children, Hackney Road, Bethnal Green.**—This hospital more particularly serves the districts of North-East, East, and North London, but is open to children from all parts. Within a mile and a half radius there is a population (mostly industrial and labouring) of over 500,000. It has 134 beds in London, and thirty at a branch at Bexhill (the "Little Folks" Home). Its expenditure, under present circumstances, is over £16,000 a year (hospital and seaside branch). Its income from endowments is about £500 a year. Last year 40,094 children were treated in the out-patient department, and the attendances of patients numbered 87,582. These figures are larger than those of any previous years. The demands upon the in-patient accommodation are now greater than ever before. A great many serious cases are unavoidably refused admission for want of room. The hospital has no funds in hand, and must shortly become heavily involved in debt unless additional support can be obtained. It specially requires £2,000 before the end of June to meet outstanding accounts. Contributions should be sent to the Secretary, Mr. T. Glenton-Kerr, at the hospital.

**Royal Ear Hospital, Dean Street, Soho, W.**—For over a century this hospital has been the means of conferring great benefits to the sick poor suffering from deafness and all diseases of the ear. It is unendowed, and a building debt of £3,000 hangs like a millstone round its neck. Beds were gratuitously placed at the disposal of the Admiralty for naval men suffering from deafness or ear injuries. It is hoped that the numerous friends of the hospital, bearing these facts in mind, will give it increased support this year.

**Royal Free Hospital, Gray's Inn Road, W.C.**—2,524 patients were admitted to the wards and more than 114,000 attendances of out-patients are recorded for the past year. For the latter improved accommodation is an urgent necessity, and towards the cost of this extension an appeal is made for £50,000. The income during 1914 was insufficient to meet the necessary expenditure by upwards of £5,000. To make up this deficit and to provide for ordinary maintenance during the current year increased support is urgently needed.

**Royal Hospital for Diseases of the Chest.**—This hospital is in a serious financial position, and for several years past the expenditure has been considerably in excess of its income. The annual expenditure averages £7,500; the more or less reliable income amounts to £3,700, leaving an annual deficit of £3,800. The result is that the hospital is indebted to its bankers to the extent of £7,700, and during the last four years there has been a falling off in annual subscriptions of upwards of £400. The outlook is, therefore, serious, and the committee appeal urgently for funds. A number of soldiers suffering from diseases of the chest are under treatment.

**Royal London Ophthalmic Hospital, City Road, E.C.**—This hospital is well known to the suffering poor as Moorfields Eye Hospital, and every day it relieves about 400 out-patients and over 100 in-patients. It is famous throughout the world as a school of ophthalmic surgery, and thus benefits rich and poor alike. For four years in succession the income of the hospital has fallen short of the expenditure, and the hospital needs immediate and generous help. The committee have placed at the disposal of the authorities thirty beds for wounded and invalided sailors and soldiers. In the out-patient department there are about 200 attendances of soldiers every month.

**Royal National Hospital for Consumption, Ventnor.** Funds are urgently needed for the maintenance of the large number of patients. The annual expenses are about £14,000, but the annual subscriptions show a decrease of £129 10s. The board would like to erect a special building where an installation of *x*-ray and other apparatus might be placed, and also to

[*Continued on p.* 24.

### A YEAR'S WORK IN THE MEDICAL CHARITIES. [Continued from p. 19

### STRATFORD AND EAST-END DISTRICT.

**Comprising Bethnal Green, Tower Hamlets, West Ham, Whitechapel, Hackney, Stepney, Limehouse, Poplar, and the East.**

| No. of Beds. | No. of Beds Daily Occupied. | HOSPITALS. | In-patients | Out-patient Attendances. | Total Expenditure. | Income. Charitable. | Income. Proprietary. | Income. Patients' Payments. | Total Income. | Legacies not included in preceding column. |
|---|---|---|---|---|---|---|---|---|---|---|
| | | | | | £ | £ | £ | £ | £ | £ |
| 922 | 800 | London ... ... ... ... | 17,539 | 571,670 | 137,519 | 61,428 | 37,523 | 7,302 | 106,253 | 27,291 |
| 50 | 40 | Mildmay Mission Hospital ... | 536 | 38,807 | 4,687 | 2,704 | 1,077 | 248 | 4,029 | ... |
| 30 | 20 | Mildmay Memorial ... ... | 250 | ... | 2,093 | 868 | 1,143 | 208 | 2,219 | ... |
| 103 | 79 | Poplar ... ... ... ... | 1,570 | 67,704 | 10,168 | 8,892 | 2,406 | 274 | 11,572 | 584 |
| 46 | 36 | Walthamstow, &c. ... ... | 533 | 14,061 | 2,279 | 1,952 | 246 | 115 | 2,313 | 10 |
| 140 | 102 | West Ham, &c. ... ... ... | 1,579 | 131,554 | 12,556 | 12,285 | 939 | 25 | 13,249 | 2,901 |
| 175 | 162 | City of London for Dis. of the Chest | 859 | 31,193 | 15,440 | 9,209 | 1,326 | 2,821 | 13,356 | 4,783 |
| 126 | 113 | East London for Children ... | 1,774 | 81,745 | 11,236 | 7,728 | 1,499 | ... | 9,227 | 1,125 |
| 43 | 41 | St. Mary's, Plaistow, for Children | 679 | 42,211 | 4,594 | 3,888 | 446 | 173 | 4,507 | 178 |
| 33 | 25 | East End Mothers' Home ... | 553 | 22,382 | 2,658 | 1,098 | 1,278 | 234 | 2,610 | 500 |
| 20 | 11 | Plaistow Maternity ... ... | 305 | ... | 540 | 359 | 43 | 159 | 561 | 57 |
| 25 | 22 | Canning Town Cottage ... ... | 304 | 15,725 | 2,033 | 1,143 | 499 | 387 | 2,029 | ... |
| 14 | 8 | Passmore Edwards Cottage, T'lb'ry | 146 | 1,914 | 870 | 731 | 83 | 8 | 822 | ... |
| 19 | 11 | East Ham Cottage ... ... | 150 | 14,971 | 980 | 749 | 105 | 55 | 909 | ... |
| 1,746 | 1,470 | | 26,777 | 1,033,937 | 207,653 | 113,034 | 48,613 | 12,009 | 173,656 | 37,429 |
| | | DISPENSARIES. | | | | | | | | |
| ... | ... | All Saints', Buxton Street ... | ... | 2,538 | 96 | 95 | ... | ... | 95 | ... |
| ... | ... | Eastern ... ... ... ... | ... | 33,895 | 939 | 196 | 275 | 432 | 903 | 23 |
| ... | ... | London ... ... ... ... | ... | 3,142 | 431 | 80 | 261 | ... | 341 | ... |
| ... | ... | Mildmay Medical Mission ... | ... | 5,901 | 162 | 92 | ... | 28 | 120 | ... |
| ... | ... | Queen Adelaide's... ... ... | ... | 12,141 | 537 | 427 | 304 | ... | 731 | 1,444 |
| ... | ... | Tower Hamlets ... ... ... | ... | 10,013 | 512 | 278 | 26 | 72 | 376 | ... |
| ... | ... | Whitechapel Provident ... ... | ... | 5,527 | 410 | 111 | ... | 323 | 434 | ... |
| 1,746 | 1, 70 | | 26,777 | 1,107,094 | 210,740 | 114,313 | 49,479 | 12,864 | 176,656 | 38,896 |

### KENSINGTON AND WEST DISTRICT.

**Comprising Kensington, Paddington, Bayswater, Kilburn, Chelsea, Brompton, Fulham, Hammersmith, Chiswick, Brentford, Acton, Ealing, &c.**

| No. of Beds. | No. of Beds Daily Occupied. | HOSPITALS. | In-patients | Out-patient Attendances. | Total Expenditure. | Income. Charitable. | Income. Proprietary. | Income. Patients' Payments. | Total Income. | Legacies not included in preceding column. |
|---|---|---|---|---|---|---|---|---|---|---|
| | | | | | £ | £ | £ | £ | £ | £ |
| 334 | 315 | St. George's ... ... ... | 4,744 | 97,973 | 41,680 | 11,994 | 12,210 | 35 | 24,239 | 11,262 |
| 305 | 249 | St. Mary's ... ... ... ... | 4,068 | 117,066 | 31,343 | 11,702 | 8,633 | 326 | 20,661 | 25,126 |
| 160 | 142 | West London ... ... ... | 2,742 | 147,655 | 17,473 | 13,892 | 1,418 | ... | 15,310 | 2,140 |
| 481 | 447 | Hospital for Consumption ... | 1,721 | 30,220 | 41,239 | 13,924 | 3,450 | 15,074 | 32,448 | 9,847 |
| 40 | 35 | Belgrave, for Children ... ... | 731 | 43,944 | 3,423 | 3,692 | 699 | 92 | 4,483 | ... |
| 81 | 78 | Cheyne, for Sick & Incurable Chldn | 37 | ... | 4,365 | 1,496 | 1,486 | 550 | 3,532 | 2,426 |
| 18 | 16 | Kensington, General .. ... | 309 | 28,808 | 2,843 | 2,521 | 15 | 417 | 2,953 | 20 |
| 13 | 9 | Kensington, for Children .. | 160 | 13,044 | 1,100 | 935 | 166 | ... | 1,101 | 50 |
| 46 | 36 | Paddington Green, for Children | 644 | 52,191 | 5,512 | 3,055 | 955 | 316 | 4,326 | 748 |
| 168 | 143 | Victoria, for Children ... ... | 1,376 | 60,478 | 10,720 | 6,476 | 2,359 | 505 | 9,340 | 536 |
| 50 | 42 | Chelsea, for Women ... ... | 744 | 8,277 | 5,887 | 2,953 | 895 | 892 | 4,740 | 100 |
| 102 | 77 | Cancer ... ... ... ... | 711 | 13,648 | 18,855 | 3,825 | 7,916 | ... | 11,741 | 50,492 |
| 145 | 120 | Female Lock ... ... ... | 343 | ... | 7,193 | 4,565 | 34 | 1,744 | 6,343 | 218 |
| 26 | 25 | Banstead Surgical Home ... | 97 | ... | 823 | 237 | 29 | 528 | 794 | ... |
| 30 | 20 | Acton Cottage ... ... ... | 292 | 6,121 | 1,544 | 1,450 | 87 | 138 | 1,675 | ... |
| 40 | 26 | Ealing Cottage ... ... ... | 465 | ... | 2,551 | 1,972 | 207 | 507 | 2,686 | 26 |
| 16 | 10 | Epsom and Ewell Cottage ... | 187 | ... | 854 | 533 | 45 | 193 | 771 | 100 |
| 27 | 18 | Hounslow Cottage ... ... | 274 | 5,013 | 1,263 | 607 | 196 | 360 | 1,163 | 100 |
| 40 | 31 | Reigate and Redhill Cottage ... | 637 | ... | 2,565 | 2,413 | 134 | 234 | 2,781 | 2,172 |
| 24 | 20 | Teddington Cottage ... ... | 355 | ... | 1,202 | 945 | 145 | 49 | 1,139 | ... |
| 29 | 19 | Wimbledon Cottage ... ... | 363 | ... | 1,464 | 1,304 | 69 | 328 | 1,701 | 1,000 |
| 30 | 26 | Wimbledon, Nelson Hospital ... | 409 | 900 | 1,622 | 1,234 | 128 | 358 | 1,720 | 500 |
| 2,205 | 1,904 | | 21,409 | 625,338 | 205,521 | 91,725 | 41,276 | 22,646 | 155,647 | 106,863 |
| | | DISPENSARIES. | | | | | | | | |
| ... | ... | Brompton Provident ... ... | ... | 2,391 | 234 | 56 | ... | 158 | 214 | ... |
| ... | ... | Kilburn, Maida Vale ... ... | ... | 4,059 | 404 | 277 | 25 | ... | 302 | ... |
| ... | ... | Kilburn Provident ... ... | ... | 9,507 | 1,114 | 101 | 14 | 1,009 | 1,124 | ... |
| ... | ... | Notting Hill Provident ... ... | ... | 5,241 | 504 | 82 | 26 | 424 | 532 | ... |
| ... | ... | Paddington Provident ... ... | ... | 5,469 | 378 | 102 | 3 | 184 | 289 | 150 |
| ... | ... | Royal Pimlico Provident... ... | ... | 8,120 | 448 | 243 | 90 | 78 | 411 | ... |
| ... | ... | Westbourne Provident ... ... | ... | 2,413 | 231 | 47 | 5 | 196 | 248 | ... |
| 2,205 | 1,904 | | 21,409 | 662,538 | 208,834 | 92,633 | 41,439 | 24,695 | 158,767 | 107,013 |

### NEWINGTON AND SOUTH DISTRICT.

**Comprising Battersea, Wandsworth, Tooting, Balham, Streatham, Brixton, Lambeth, Newington, Southwark, Bermondsey, Camberwell, Greenwich, Deptford, Lewisham, Blackheath, Woolwich, &c.**

| No. of Beds. | No. of Beds Daily Occupied. | Hospitals. | In-patients. | Out-patient Attendances. | Total Expenditure. | Income. | | | Total Income. | Legacies not included in preceding column. |
|---|---|---|---|---|---|---|---|---|---|---|
| | | | | | | Charitable. | Proprietary. | Patients' Payments. | | |
| | | | | | £ | £ | £ | £ | £ | £ |
| 644 | 523 | Guy's ... ... ... ... | 9,408 | 474,896 | 85,973 | 14,801 | 50,122 | 5,260 | 70,183 | 29,447 |
| 775 | 332 | King's College ... ... ... | 5,981 | 120,212 | 41,336 | 10,393 | 3,460 | 9,347 | 23,200 | 21,722 |
| 18 | 10 | Phillips' Memorial Homœopathic | 145 | 1,836 | 1,178 | 568 | 294 | 316 | 1,178 | 1,090 |
| 66 | 59 | Miller ... ... ... ... | 953 | 90,450 | 8,319 | 8,393 | 1,803 | 244 | 10,440 | 1,302 |
| 60 | 36 | St. John's, Lewisham ... ... | 491 | 7,112 | 3,877 | 1,959 | 546 | 300 | 2,805 | 2,563 |
| 592 | 531 | St. Thomas's ... ... ... | 9,158 | 249,427 | 83,121 | 2,601 | 64,435 | 2,151 | 69,187 | 69,894 |
| 306 | 226 | Seamen's ... ... ... ... | 2,146 | 86,470 | 26,703 | 20,496 | 3,553 | 1,490 | 25,539 | 2,030 |
| 60 | 49 | Bolingbroke Hospital ... ... | 905 | 32,060 | 6,094 | 2,650 | 1,002 | 837 | 4,489 | 2,700 |
| 76 | 53 | Evelina, for Children ... ... | 1,017 | 52,911 | 8,016 | 2,450 | 4,121 | 331 | 6,902 | 463 |
| 57 | 37 | Home for Sick Children... ... | 338 | 3,166 | 2,269 | 1,257 | 254 | 487 | 1,998 | 30 |
| 36 | 31 | General Lying-In... ... ... | 819 | 9,229 | 5,555 | 2,691 | 3,453 | ... | 6,144 | ... |
| 50 | 28 | Clapham Maternity & Dispensary | 563 | 8,111 | 2,143 | 446 | 880 | 994 | 2,320 | ... |
| 106 | 84 | Royal Waterloo ... ... ... | 1,098 | 39,781 | 8,589 | 5,628 | 1,888 | ... | 7,516 | 1,768 |
| 42 | 34 | Royal Eye ... ... ... ... | 689 | 64,982 | 5,549 | 1,496 | 931 | 1,043 | 3,470 | 2,564 |
| 28 | 14 | Beckenham Cottage ... ... | 250 | 741 | 1,286 | 883 | 31 | 319 | 1,233 | ... |
| 26 | 16 | Blackheath Cottage ... ... | 198 | 4,284 | 1,576 | 1,122 | 59 | 255 | 1,436 | 77 |
| 42 | 27 | Bromley Cottage ... ... ... | 396 | 185 | 2,350 | 1,201 | 603 | 538 | 2,342 | ... |
| 28 | 17 | Chislehurst, &c., Cottage ... | 283 | ... | 1,746 | 586 | 230 | 635 | 1,451 | 50 |
| 22 | 9 | Eltham Cottage ... ... ... | 176 | ... | 1,063 | 574 | 110 | 214 | 898 | ... |
| 12 | 7 | Sidcup Cottage ... ... ... | 146 | 451 | 630 | 300 | 137 | 184 | 621 | ... |
| 34 | 29 | Livingstone Cottage ... ... | 407 | ... | 1,440 | 1,330 | 20 | 125 | 1,475 | ... |
| 23 | 16 | Victoria Hospital, Kingston ... | 303 | ... | 1,162 | 661 | 246 | 201 | 1,108 | 200 |
| 14 | 9 | Woolwich Home for Mothers and Babies ... ... ... ... | 174 | 2,447 | 872 | 776 | 45 | 332 | 1,153 | ... |
| 14 | 10 | Woolwich Cottage ... ... | 140 | ... | 1,030 | 295 | 1,050 | 147 | 1,492 | 11 |
| 3,131 | 2,187 | | 36,184 | 1,248,751 | 301,877 | 83,557 | 139,273 | 25,750 | 248,580 | 135,911 |
| | | **Dispensaries.** | | | | | | | | |
| ... | ... | Battersea Provident ... ... | ... | 91,000 | 3,859 | 164 | 49 | 3,681 | 3,894 | ... |
| ... | ... | Brixton, &c. ... ... ... | ... | 12,498 | 616 | 371 | 45 | 78 | 494 | ... |
| ... | ... | Camberwell Provident ... ... | ... | 57,405 | 1,149 | 248 | 163 | 645 | 1,056 | ... |
| ... | ... | Clapham ... ... ... ... | ... | 6,732 | 380 | 148 | 86 | 168 | 402 | 25 |
| ... | ... | East Dulwich Provident ... | ... | 16,768 | 943 | 79 | 23 | 863 | 965 | ... |
| ... | ... | Forest Hill Provident ... ... | ... | 14,191 | 545 | 168 | 18 | 346 | 532 | ... |
| ... | ... | Greenwich Provident ... ... | ... | 1,440 | 505 | 46 | 10 | 430 | 486 | ... |
| ... | ... | Royal South London ... ... | ... | 5,488 | 435 | 284 | 24 | 2 | 310 | ... |
| ... | ... | South Lambeth, &c. ... ... | ... | 2,556 | 357 | 113 | 63 | 60 | 236 | ... |
| ... | ... | Wandsworth Common ... ... | ... | 2,039 | 193 | 11 | ... | 182 | 193 | ... |
| ... | ... | Woolwich, &c., Provident ... | ... | 14,149 | 676 | 37 | 4 | 653 | 694 | ... |
| 3,131 | 2,187 | | 36,184 | 1,473,017 | 311,535 | 85,226 | 139,758 | 32,858 | 257,842 | 135,936 |

### THE MEDICAL CHARITIES OF LONDON.—A Summary of the Work Done in 1914.

It will be seen from the following summary that *One hundred and fifty-two thousand three hundred and sixty-two* patients were admitted into the Voluntary Hospitals and Medical Charities of London during the year 1914, and that the attendances in the Out-Patient Departments and Dispensaries numbered *Five million eight hundred and thirty-four thousand seven hundred and thirty-five*, at a cost of *£1,369,028*. The Ordinary Income only amounted to *£1,137,184*, leaving a deficiency of *£231,844* on the year's work. The Legacies received in 1914 amounted to *£417,088*.

| No. of Beds. | No. of Beds Daily Occupied. | Hospitals and Dispensaries. | In-patients. | Out-patient Attendances. | Total Expenditure. | Income. | | | Total Income. | Legacies not included in preceding column. |
|---|---|---|---|---|---|---|---|---|---|---|
| | | | | | | Charitable. | Proprietary. | Patients' Payments. | | |
| | | | | | £ | £ | £ | £ | £ | £ |
| 3,131 | 2,187 | Newington and South District ... | 36,184 | 1,473,017 | 311,535 | 85,226 | 139,758 | 32,858 | 257,842 | 135,936 |
| 1,498 | 1,257 | City and East Central District... | 20,218 | 886,216 | 191,571 | 72,553 | 85,005 | 7,081 | 164,639 | 25,635 |
| 1,018 | 779 | Westminster District ... ... | 11,103 | 450,665 | 106,905 | 63,468 | 18,717 | 21,859 | 104,044 | 23,671 |
| 2,102 | 1,775 | St. Marylebone and West Central District ... ... ... | 22,505 | 717,139 | 210,055 | 98,866 | 44,176 | 23,929 | 166,971 | 65,037 |
| 2,205 | 1,904 | Kensington and West District ... | 21,409 | 662,538 | 208,834 | 92,633 | 41,439 | 24,695 | 158,767 | 107,013 |
| 1,480 | 1,175 | Islington & North-West District | 14,166 | 538,066 | 129,388 | 72,632 | 17,869 | 17,764 | 108,265 | 20,900 |
| 1,746 | 1,470 | Stratford and East-End District | 26,777 | 1,107,094 | 210,740 | 114,313 | 49,479 | 12,864 | 176,656 | 38,896 |
| 13,180 | 10,547 | | 152,362 | 5,834,735 | 1,369,028 | 599,691 | 396,443 | 141,050 | 1,137,184 | 417,088 |

### Hospitals and their Special Needs.

[Continued from p. 21.

institute a department of research. A tower and spire to complete the chapel is much desired. Funds and new annual subscribers for these purposes are earnestly needed.

**Royal National Sanatorium for Consumption, etc., Bournemouth.**—It shows the widespread usefulness of this institution that of the patients admitted in 1914, 411 came from no fewer than thirty-one different counties in England, two from Wales, and one (a war refugee) from Antwerp. But the committee draw attention to the fact that voluntary contributions have diminished, and are £225 less than the previous year, while expenditure has unavoidably increased.

**Royal Westminster Ophthalmic Hospital, King William Street, Strand, W.C.**—Increased support is needed to meet the growing claims upon this institution. Expenses of maintenance tend constantly to increase with the advance of therapeutical science and appliances, and are still further enhanced by the special exigencies of the moment, and although the committee's appeals for support have been responded to, the ordinary income was not sufficient to meet the expenses of the charity by £222. Ten beds have been reserved for the use of invalided soldiers suffering from injuries or diseases of the eye, and the hospital is daily attending to numbers of recruits for the new Armies and Territorial Forces temporarily below the standard of fitness required.

**St. George's Hospital, S.W.**—Although some years back the annual subscriptions of this charity were £8,000, they have been gradually reduced till last year only £4,602 was derived from this source. The ordinary expenditure in 1914 exceeded the ordinary income by £15,710, which had to be met by the sale of stock, by an overdraft at the bank, and by using up legacies received. With the cost of provisions and drugs, etc., being higher than in 1914, it is feared that the excess of expenditure in 1915 will be much higher. While ministering to the sick poor as usual, several beds have been allocated for the reception and treatment of the sick and wounded soldiers from the Front, of whom 105 were admitted up to December 31, 1914. The house committee most urgently appeal to the charitable to help them, so that they can maintain the hospital in its full efficiency.

**St. John's Hospital for Diseases of the Skin, Leicester Square, W.C.** — For over fifty years St. John's has been doing steady and excellent work, and at the present time some 300 in-patients are treated annually, and in round figures 8,000 out-patients, making a total of about 40,000 attendances. The hospital needs special help for renewing its electrical plant—cost, about £250. Since August, 258 sailors and soldiers and thirty-two refugees have been treated free of charge, but a large debt remains unpaid, and donations have practically stopped.

**St. John's Hospital, Morden Hill, Lewisham, S.E.**—During the past year the utmost economy consistent with efficiency was practised, and resulted in a decrease in the total ordinary expenditure. At the same time, the numbers of in- and out-patients have increased, and the amount received from donations was £500 less than in the previous year. Expenses have necessarily increased, and the upkeep of the hospital is practically dependent upon donations and subscriptions. The hospital placed forty beds at the disposal of the Government for wounded soldiers. Up to December 31, 1914, 112 wounded British and Belgian soldiers had been treated, and forty are at present in the hospital. The wounded were at first received free of cost, but since January payment (in part only) has been applied for from the Government.

**St. Mark's Hospital, City Road, E.C.**—Before the war the committee had decided, at a cost of over £1,000, to build a special women's ward for cancer patients, also additional accommodation for the nursing staff, but unless the charitable public see their way to support this effort to cope with the ever-increasing cases of rectal cancer this scheme must become at present impossible. Twelve beds have been reserved for wounded soldiers.

**St. Mary's Hospital, Paddington, W.**—This hospital has 305 beds, and its work costs £30,000 a year. Regular sources of income yield only one half of this, and for the remaining £15,000 it is dependent on voluntary contributions. Distinguished among hospitals for the services it is rendering to the country in the present terrible crisis, St. Mary's is yet suffering heavily financially through the war. In addition to nursing sick and wounded soldiers from the Front, St. Mary's has manufactured and supplied not only our own but all the Allied Armies with anti-typhoid and other vaccines. Several million doses have been supplied, and this work has been acknowledged by the Army Council as "of great practical value to the troops in the field as well as to the State." Similar testimony has been borne by the Belgian, French, Russian, and Serbian authorities. It is common knowledge that the Allied Armies in the Western battlefield have enjoyed a degree of immunity from typhoid fever which has deprived war of one of its terrors, and this is due in a great degree to work done in St. Mary's Hospital.

**St. Monica's Home Hospital for Sick Children, 16 Brondesbary Park, N.W.**—For over forty years this hospital has carried on its beneficent work, and until the past year, free from debt. During 1914, however, there was a considerable falling-off in the receipts, from donations chiefly, and at the end of the year there was a deficiency of £311. To meet this deficiency the committee of management have had to raise a loan, but they are most anxious to pay it back as soon as possible, and appeal to friends and lovers of children to help them to do so, and also to obtain additional support to meet the increased expenses of maintenance during the current year. A few Belgian children have been received as patients.

**St. Peter's Hospital for Stone, etc., Henrietta Street, W.C.**—During the past year, to comply with the requirements of the L.C.C., two external iron staircases, as a means of escape in case of fire, have been erected at a cost of £640. King Edward's Hospital Fund contributed the sum of £200, but the rest had to be met by a loan. Decreasing contributions and increasing costs of commodities make the task of financing the hospital one of great anxiety. Twelve beds were reserved for the use of wounded soldiers, though these have not so far been occupied; but patients have been treated to enable them to join the Army or proceed to the Front, and many of the staff and nurses have joined the Forces.

[Continued on p. 26.

### THE VOLUNTARY HOSPITALS' BUDGET.

Hospital Sunday has again come round, bringing with it the necessity for considering ways and means, but this year the circumstances of the time we are living in are of a totally different character from those which were current when we discussed the 1914 budget of the Metropolitan Hospitals and Dispensaries in our last Hospital Sunday Special Number. Then we appealed for gifts out of the fullness of the purses of the charitable; now, when everyone is struggling under the ever-increasing burden of the war, our appeal to them must be: Out of your smaller spending power make your gifts to the medical charities at least what they used to be, for the necessities of these charities are infinitely greater to-day than they were a year ago. With still greater emphasis than ever, too, do we appeal to those who have not in the past extended their gifts to this form of charity. Now is their opportunity to come to the assistance of those who hitherto have shouldered the burdens of the voluntary charities, and, by providing a new field from which hospital managers can look for revenue, supplement the efforts of their fellow-citizens who have kept up the voluntary system for the benefit of all.

The ordinary expenditure of the Metropolitan Voluntary Hospitals and Dispensaries is increasing by about £40,000 a year. In 1911 it was about £1,215,000; in 1912, about £1,255,000; and in 1913, about £1,295,000. In 1915, therefore, it will certainly be, on a very conservative estimate, not less than £1,300,000, strive though the managers may to cut down expenses in every possible direction. And to meet this expenditure the hospitals and dispensaries have to rely upon three main sources of revenue.

First and most important of these are the gifts of the charitable for which we are now appealing, such as subscriptions, donations, entertainments, amounts raised by the King's Fund and the Hospital Sunday and Saturday Funds, and other receipts of this character. This source of revenue, together with the payments made by patients for treatment received, supplies over half the amount required for the upkeep of the hospitals, and may be taken as worth about £750,000 a year. Whether it will reach that figure in 1915 is a matter which now depends to a great extent upon the possibility of the old supporters continuing to give as they have done in the past, and upon new givers flocking in to fill up the gaps which may be caused by smaller subscriptions and donations being sent by former givers. £750,000 is the equivalent of 11s. 6d. towards every sovereign of the anticipated expenditure of £1,300,000.

Secondly, there is the income from investments and property. The value of this is increasing by about £10,000 a year, and should continue to do so. The amount received in 1913 was £350,000; so that in 1915 about £370,000 should be received under this head, or about 5s. 8d. towards each sovereign of the estimated expenditure for that year.

These two fairly certain sources of income may therefore be looked on as providing 17s. 2d. towards every £1 to be expended, and there only remains one other quarter from which revenue can be derived, viz. the gifts left to hospitals in the shape of legacies. These naturally vary in amount from year to year, having been during the last ten years as low as £186,000 (in 1906), and as high as £424,000 (in 1913). The average for the ten years (1904-1913) has been £320,000. Assuming legacies in 1915 reach the average of the last ten years, it will be necessary to take £180,000, or nearly three-fifths, of the legacies to be expected, in order to meet the estimated expenditure for the year. In reality, however, legacies should be looked upon as of the nature of extraordinary income, and should be utilised to assist in meeting the expenditure required for rebuilding, for improvements necessitated by the rapid march of science, or for extensions where overcrowding has become apparent, all of which items of extraordinary expenditure have now to be met by special appeals to those who already give largely.

Extraordinary expenditure we have entirely left out of consideration in our estimates as we are more concerned to-day with what we have to do to meet the ordinary every-day needs of the hospitals. It may, perhaps, be as well, however, to mention here that a further sum of from £200,000 to £300,000 is found each year under this head to meet the special needs which arise from time to time in looking after the care of the sick of the Metropolis.

In conclusion, therefore, we ask the old supporters of the Metropolitan Hospitals and Dispensaries to give this year, as nearly as possible, as liberally as they have done in the past; and we call on all those who have not hitherto given to remember what the voluntary hospitals have done, and are doing, for the entire community, and to become supporters of them at a time when it seems possible these charities may have to suffer in consequence of the heavy calls being made on the rest of the community.

Table Showing the Sources of the Income of the Metropolitan Hospitals and Dispensaries in the Ten Years 1904 to 1913.

| Year | From the Living | | From the Dead | | | | Total Income |
|---|---|---|---|---|---|---|---|
| | Subscriptions, Donations, Patients' payments, etc. | Percentage of Total | Investments | Percentage of Total | Legacies | Percentage of Total | |
| | £ | | £ | | £ | | £ |
| 1904 | 603,425 | 50 | 258,567 | 22 | 319,303 | 28 | 1,181,295 |
| 1905 | 610,130 | 49 | 264,790 | 21 | 373,914 | 30 | 1,248,834 |
| 1906 | 635,377 | 58 | 272,704 | 25 | 186,284 | 17 | 1,094,365 |
| 1907 | 653,320 | 48 | 296,218 | 22 | 407,918 | 30 | 1,357,456 |
| 1908 | 712,461* | 56 | 290,491 | 23 | 265,493 | 21 | 1,268.445 |
| 1909 | 701,458 | 57 | 294,206 | 24 | 240,523 | 19 | 1,236,187 |
| 1910 | 703,841 | 50 | 309,320 | 22 | 394,478 | 28 | 1,407,639 |
| 1911 | 738,622 | 53 | 329,253 | 24 | 322,949 | 23 | 1,390,824 |
| 1912 | 741,439 | 55 | 338,425 | 25 | 272,090 | 20 | 1,351,954 |
| 1913 | 845,908* | 52 | 349,295 | 22 | 424,339 | 26 | 1,619,542 |

* Includes £72,565 and £103,970, the amounts received by the London Hospital in 1908 and 1913 respectively as the result of its Quinquennial Appeals in those years.

### Hospitals and their Special Needs.

[Continued from p. 24.

**Seamen's Hospital Society (Dreadnought and Albert Dock Hospitals), Greenwich, S.E.**—The work of the Seamen's is universal; it is not confined to any particular country, and to maintain its 300 beds for the sailors of all nations new subscriptions and donations are urgently needed. By an arrangement with the Admiralty and War Office large numbers of wounded from the war are received into the wards of both hospitals.

**Victoria Hospital for Children, Chelsea.**—During the latter part of 1914 the funds of this hospital decreased considerably owing to the many appeals in connection with the war, consequently there was a deficit of £634 that had to be brought forward into the new year. The increased cost of nearly every article of consumption must necessarily increase the expenses this year, and the committee therefore earnestly appeal for assistance. Two wards were given to the War Office for soldiers invalided from the Front, and over 200 have been admitted for treatment, in addition to 1,309 children.

**West End Hospital, Welbeck Street, W.**—Owing to the war a much needed rebuilding scheme has been temporarily suspended. New subscribers are wanted, as the ordinary work costs £6,500 per annum, while the income from invested property amounts to £1,300, and there is a debt of £3,000 to wipe out. In addition to the usual work for civilians (which has not been curtailed) temporary wards for nerve-injured and nerve-wrecked soldiers have been opened. Many of these are very bad and tedious cases, and require lengthy treatment. The War Office contributes towards their maintenance; but this contribution has to be largely supplemented from the hospital funds, and the outlay in preparing this new accommodation has also to be met.

**West London Hospital, Hammersmith.**—The hospital serves an immense district, having an area of forty-five square miles and a population of over half-a-million. No fewer than 40,000 sick and poor in- and out-patients are treated every year. The annual expenditure amounts to £17,500, and the assured income from endowments is £600 only. Practically £17,000 has to be raised every year from voluntary sources. One effect of the war has already been felt in a serious reduction in the amount of subscriptions received. This, and the greatly enhanced cost of all commodities, render the task of the board of management in finding the necessary funds a very anxious one. The hospital is doing its best to help in the present crisis, and on the outbreak of war accommodation for 100 sick and wounded soldiers from overseas was placed at the disposal of the War Office. Between October and March 337 soldiers were received as in-patients and 650 as out-patients. It is worthy of note that out of this number not one succumbed to his ailments or injuries. Subscriptions and donations towards carrying on the West London Hospital's vast and increasing work will be most gratefully received and acknowledged.

**Westminster Hospital, London, S.W.**—The expenditure necessary to the maintenance of this the oldest hospital in London supported by voluntary contributions shows a deficit of £2,569 over income for the first quarter of the current year. If, therefore, this charity is to maintain efficiently its good work, additional support of the most generous character is immediately required. The removal of the hospital to a more commodious site is in contemplation, and as this will involve a very large expenditure, liberal contributions to the "Removal Fund" are very earnestly solicited. The total number of beds is 213, and upon the outbreak of war seventy-five of these beds were placed at the disposal of the War Office for the treatment of sick and wounded soldiers from the Front. Up to the present date 250 British and fifty-two Belgian soldiers have been treated.

### OTHER INSTITUTIONS AND THEIR SPECIAL NEEDS.

**Baldwin Brown Convalescent Home, Herne Bay.**—Although over 500 persons were received at this home last season, the applications far exceed the available accommodation. Twenty-three Belgian refugees of the industrial class were received in October. Some of these were Mechlin tapestry weavers, and the task of finding these refugees employment is a difficult one, and calls for solution.

**Convalescent Home for Poor Children, St. Leonards-on-Sea.**—Contributions are needed to the general fund to enable the work amongst the poor children, most of whose fathers are sailors or soldiers, to be carried on.

**Hostel of God (Free Home for the Dying), 29 North Side, Clapham Common, S.W.**—This charity admits sufferers from incurable diseases—chiefly of cancer and consumption—who consequently cannot be retained in hospital because nothing more can be done for them. A strong appeal is therefore made.

**Metropolitan Convalescent Institution, 14 Victoria Street, S.W.**—The maintenance of the four homes at Walton, Broadstairs, Bexhill-on-Sea, and Little Common, Bexhill, containing 586 beds in all, costs £14,000 a year, for nearly the whole of which the institution is dependent upon voluntary contributions. The Board of Management appeal very earnestly for further annual subscriptions and donations.

**St. Mary's Convalescent Home for Children, Broadstairs.**—Over 1,000 of the poorest children of the land, most of whom have never before seen the sea, come to this home yearly and are restored to health. Funds are urgently needed to renew the heating apparatus throughout the home.

## Figures and Tables

**Figure f1:**
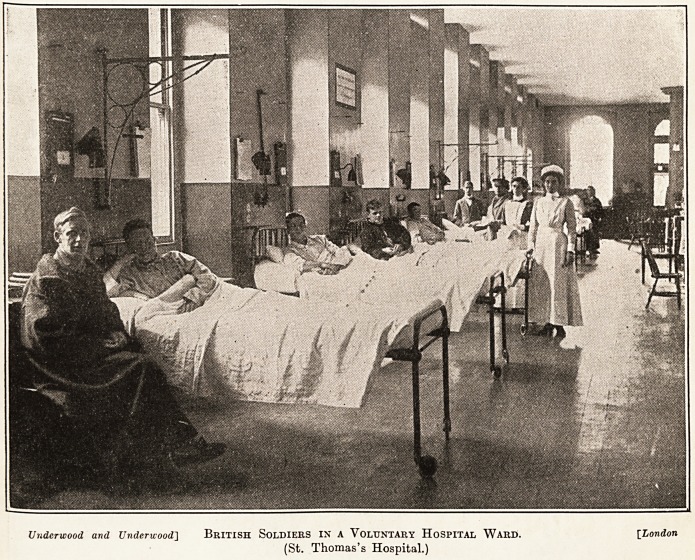


**Figure f2:**
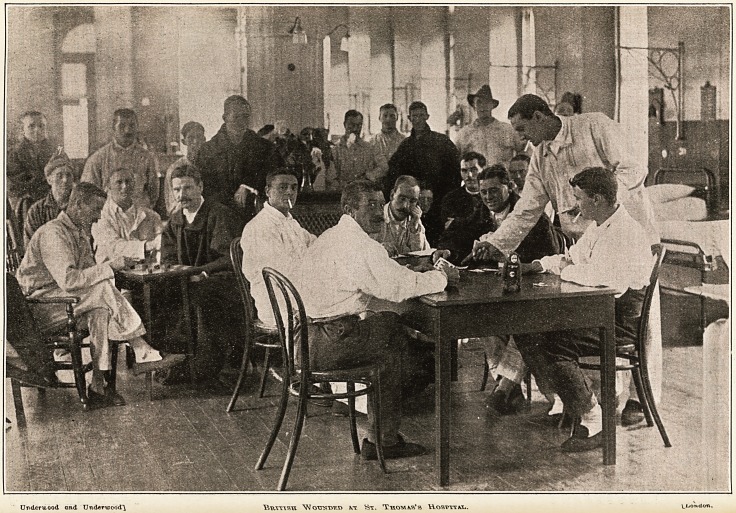


**Figure f3:**
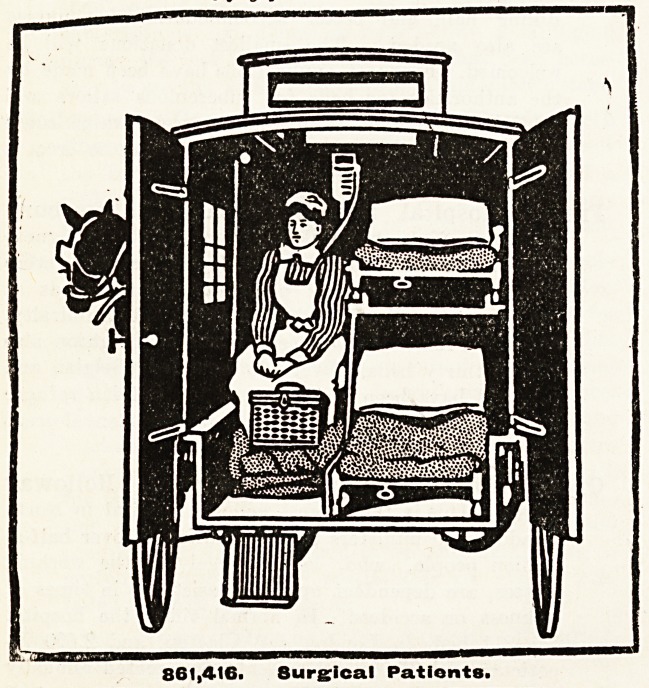


**Figure f4:**
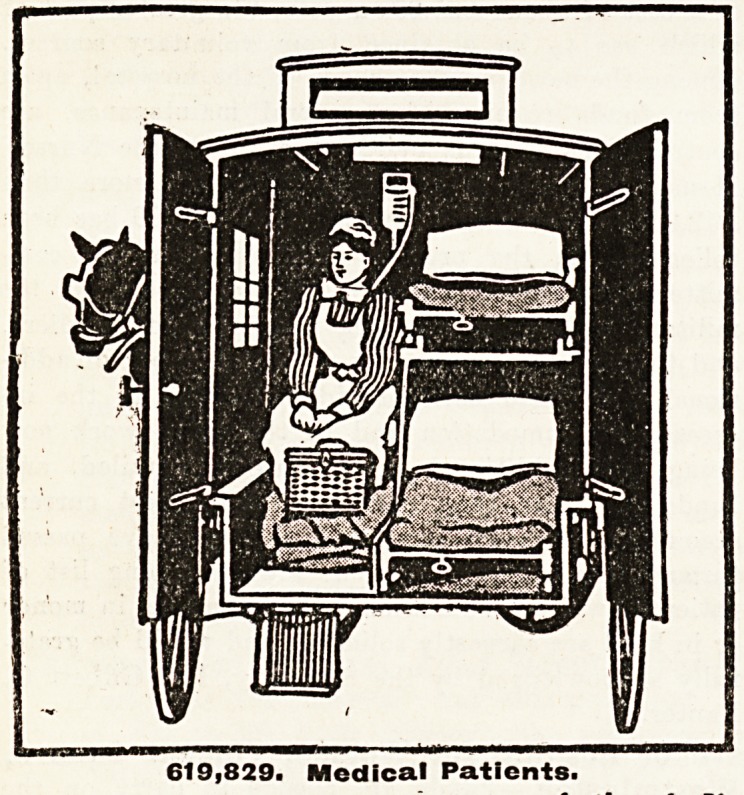


**Figure f5:**
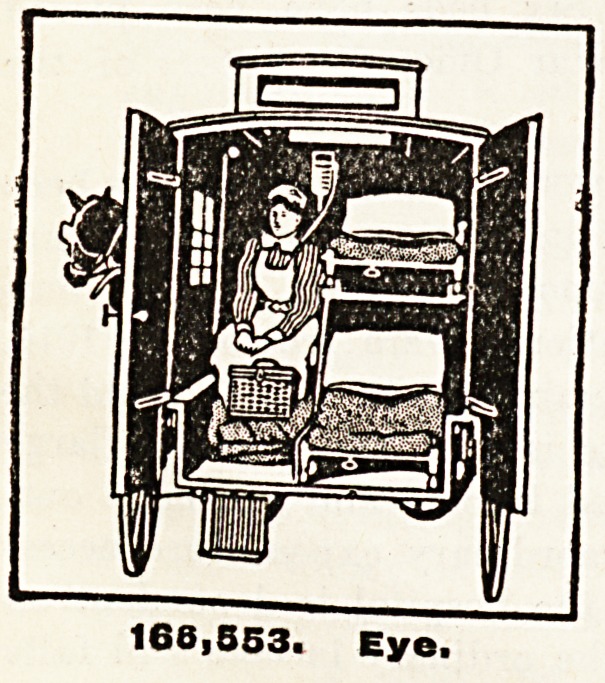


**Figure f6:**
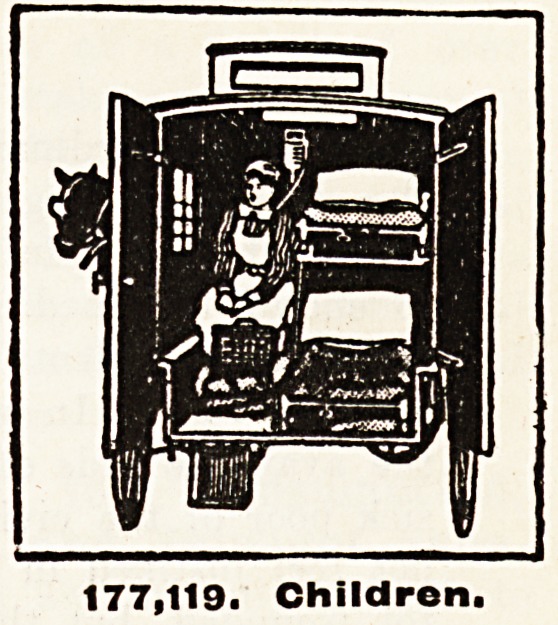


**Figure f7:**
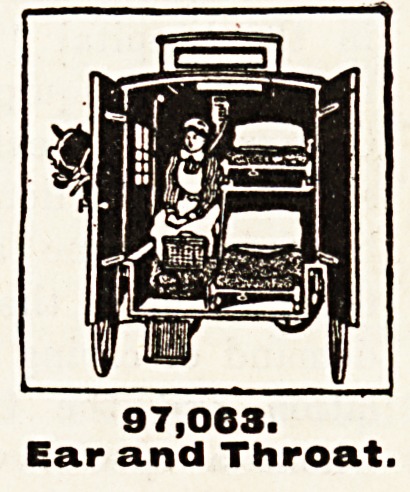


**Figure f8:**
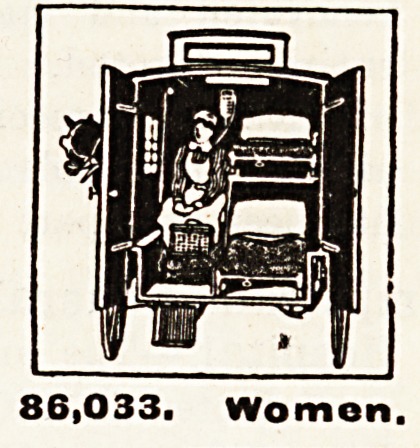


**Figure f9:**
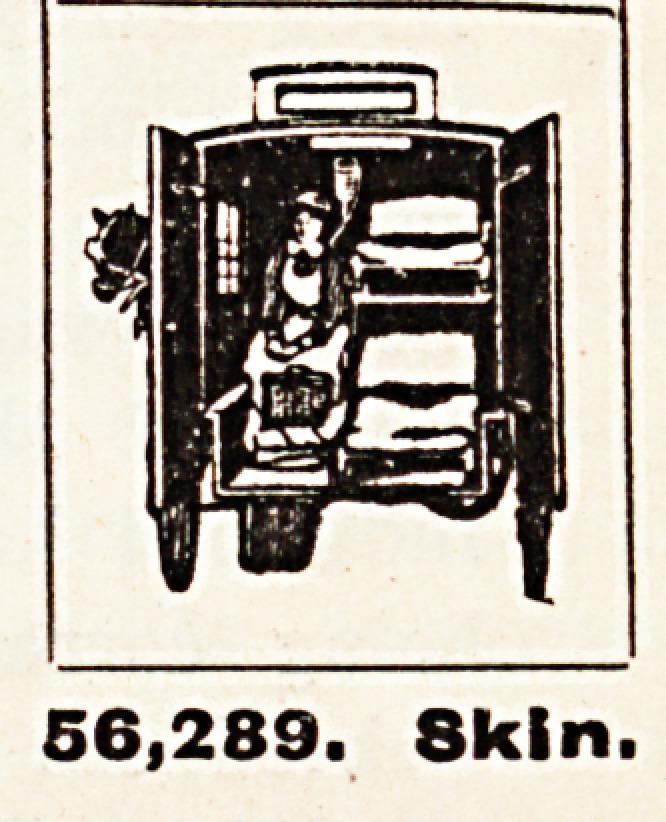


**Figure f10:**
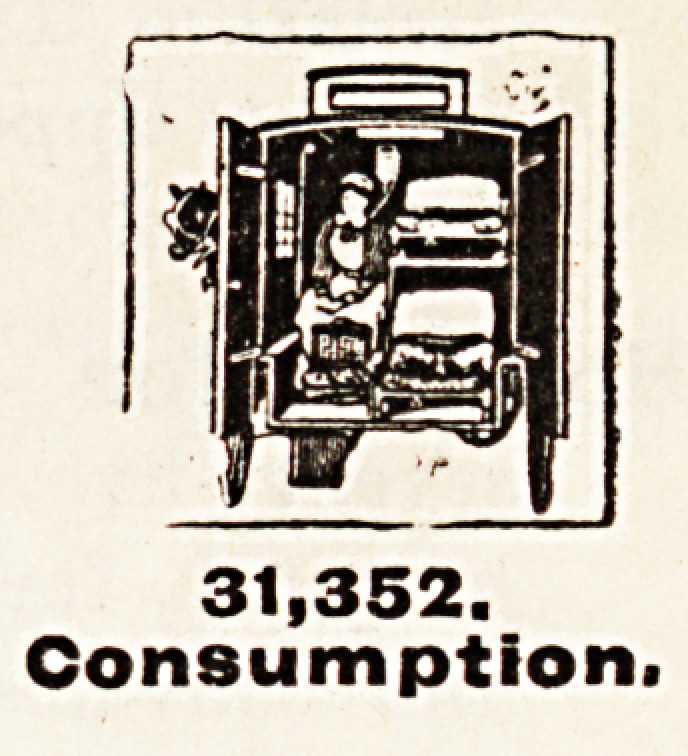


**Figure f11:**
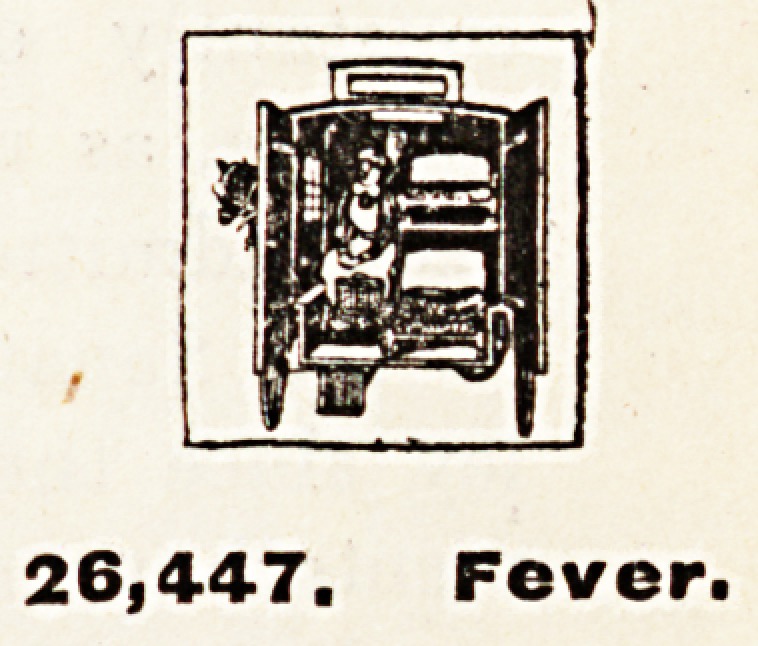


**Figure f12:**